# C11orf94/Frey is a key regulator for male fertility by controlling Izumo1 complex assembly

**DOI:** 10.1126/sciadv.abo6049

**Published:** 2022-08-12

**Authors:** Whendy Contreras, Caroline Wiesehöfer, Dora Schreier, Nadja Leinung, Petra Peche, Gunther Wennemuth, Marc Gentzel, Bernd Schröder, Torben Mentrup

**Affiliations:** ^1^ Institute of Physiological Chemistry, Technische Universität Dresden, Dresden, Germany.; ^2^ Department of Anatomy, University Hospital, University of Duisburg-Essen, Essen, Germany.; ^3^ CRISPR-Cas9 Facility, Experimental Center of the Medical Faculty Carl Gustav Carus, Technische Universität Dresden, Dresden, Germany.; ^4^ Core Facility Molecular Analysis–Mass Spectrometry, Center for Molecular and Cellular Bioengineering (CMCB), Technische Universität Dresden, Dresden, Germany.

## Abstract

Although gamete fusion represents the central event in sexual reproduction, the required protein machinery is poorly defined. In sperm cells, Izumo1 and several Izumo1-associated proteins play an essential role for this process. However, so far, the mechanisms underlying transport and maturation of Izumo1 and its incorporation into high molecular weight complexes are incompletely defined. Here, we provide a detailed characterization of the C11orf94 protein, which we rename Frey, which provides a platform for the assembly of Izumo1 complexes. By retaining Izumo1 in the endoplasmic reticulum, Frey facilitates its incorporation into high molecular weight complexes. To fulfill its function, the unstable Frey protein is stabilized within the catalytic center of an intramembrane protease. Loss of Frey results in reduced assembly of Izumo1 complexes and male infertility due to impaired gamete fusion. Collectively, these findings provide mechanistic insights into the early biogenesis and functional relevance of Izumo1 complexes.

## Teaser

The intramembrane protease–regulated Frey protein is essential for gamete fusion by controlling Izumo1 complex assembly.

## INTRODUCTION

The fusion of sperm and oocyte represents an essential step in mammalian fertilization and sexual reproduction (*1*). Following sperm capacitation within the female genital tract, the sperm acrosome reaction is triggered by a still not fully elucidated mechanism that among others might involve interaction with specific proteins within the zona pellucida (ZP) of the oocyte (*2*). As a consequence, increasing cellular calcium levels induce the fusion of the outer acrosomal membrane with the sperm plasma membrane. This process leads to both the liberation of enzymes required for efficient penetration of the ZP and the presentation of proteins required for sperm-oocyte attachment and fusion on the sperm surface (*3*). In particular, the latter two processes are dependent on the presence of Izumo1, which was initially identified as molecular target of a monoclonal antibody effectively preventing sperm-oocyte fusion (*4*). Izumo1 is a type I transmembrane protein that belongs to the immunoglobulin superfamily. Before sperm capacitation and acrosome reaction, it is located within the outer acrosomal membrane in the sperm head (*5*). Upon induction of the acrosome reaction, Izumo1 relocates to the plasma membrane of the sperm cell where it becomes accessible for interaction with the oocyte (*4*, *6*). Although sperms of Izumo1-deficient mice show no overt morphological differences to those of wild-type animals and are still capable of attaching to oocytes, they cannot fertilize these cells because of a block in gamete fusion, highlighting the essential role for Izumo1 in this process (*4*, *7*). Later, Juno was identified as the oocyte receptor for Izumo1. Comparable to the situation for Izumo1 in sperm, loss of Juno leads to female infertility and loss of sperm-oocyte fusion, revealing the essential relevance of the Izumo1-Juno interaction (*8*–*10*).

Despite the clear evidence for a function of Izumo1 and Juno in sperm-oocyte fusion, the interaction of both molecules is most likely not sufficient for induction of this process despite some conflicting evidence (*11*, *12*). Although human embryonic kidney (HEK) cells overexpressing Izumo1 efficiently bind to mouse oocytes (*13*), they are unable to fuse with these cells (*8*, *13*). Izumo1 is part of a high molecular weight (HMW) complex in sperm cells, suggesting that it might fulfill its function as part of a multiprotein complex (*14*–*16*). Over the past years, several proteins have been identified that, in addition to Izumo1, are required for gamete fusion: fertility influencing membrane protein (FIMP), sperm-oocyte fusion required 1 (SOF1), dendrocyte expressed seven transmembrane protein (DC-STAMP) domain containing 1/2 (DCST1/2), sperm acrosome membrane–associated protein 6 (SPACA6), Glioma pathogenesis-related protein 1 like 1 (GLIPR1L1), and transmembrane protein 95 (TMEM95) (*13*, *16*–*19*). With the exception of DCST1/2, for which interaction was not tested, all of these proteins physically interact with Izumo1, suggesting that they might act together in a HMW complex with Izumo1 to coordinate gamete fusion (*13*, *17*). However, how these structurally diverse proteins contribute to sperm-oocyte fusion and how their incorporation into a multiprotein complex might be facilitated in the dynamic context of a differentiating male germ cell remains elusive.

Recently, the contribution of the intramembrane-cleaving protease (I-CLiP) signal peptide peptidase-like 2c (SPPL2c) to regulation of male fertility has been demonstrated. SPPL2c is a nine–transmembrane domain (TMD) aspartic protease that is highly expressed in the spermatid stage of male germ cells but absent from mature spermatozoa (*20*, *21*). In contrast to the closely related γ-secretase that acts on a multitude of substrate molecules in a rather promiscuous way (*22*, *23*), mass spectrometric analysis of testis membranes of wild-type or SPPL2c-deficient mice only revealed two physiological substrates of SPPL2c: phospholamban (PLN) that is involved in regulation of endoplasmic reticulum (ER)–resident calcium pumps and the SNAP Receptor (SNARE)-molecule Syntaxin8 (Stx8). Proteolysis of these two proteins is connected to the regulation of calcium levels (*20*) and acrosome formation (*24*) in spermatids. However, despite showing subtle impairments in testis weight and sperm motility, SPPL2c-deficient males reveal no overt reduction in fertility when paired with wild-type females, demonstrating that SPPL2c itself is not essential for male fertility (*20*). Nevertheless, the strong and highly specific expression of SPPL2c in spermatids is indicative for potential additional functions of this protease in this cell type, which might have escaped the initial characterization of this molecule.

Here, by analyzing the so far uncharacterized murine C11orf94 homolog (C11orf94) protein, which is encoded by the *1700029I15Rik* gene, we provide insights not only into both the function of SPPL2c within the murine testis but also into the mechanism of early biogenesis of Izumo1-containing complexes. In the abovementioned mass spectrometry (MS) dataset, C11orf94 was significantly down-regulated in the testis of SPPL2c-deficient mice (*20*), suggesting that both proteins function within the same biological pathway. C11orf94 is stabilized in the catalytic center of SPPL2c to bind Izumo1 within the ER of round spermatids. This process is required for the proper assembly of Izumo1-containing complexes. In line with this important function, C11orf94-deficient male mice are infertile. In addition to revealing a previously unknown and unique mode of intramembrane protease action, these findings provide a model for how assembly of Izumo1-containing complexes can be facilitated even in a differentiating germ cell rearranging its secretory pathway.

## RESULTS

**Fig. 1.**
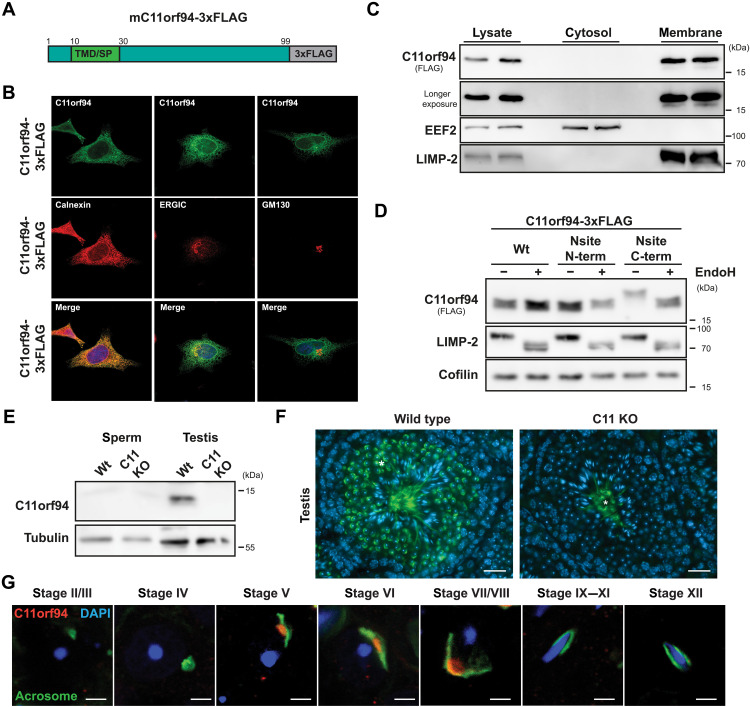
C11orf94 is a testis-specific type II ER protein. (**A**) Scheme of the wild-type C11orf94 expression construct equipped with a C-terminal 3xFLAG-tag. At the N terminus of the protein, a predicted TMD, which might also function as signal peptide (SP), is highlighted. (**B**) HeLa cells were transfected with mC11orf94-3xFLAG and analyzed by indirect immunofluorescence. Scale bars, 10 μm. (**C**) HEK cells were transfected with mC11orf94-3xFLAG, and cellular membranes were purified by ultracentrifugation. C11orf94 was detected by Western blotting; the membrane protein LIMP-2 and the cytosolic EEF-2 protein served as controls. (**D**) An artificial N- or C-terminal N-glycosylation site (Nsite, sequence NIS) was introduced to C11orf94. HEK cells transfected with the indicated constructs were lysed, and lysates were subsequently subjected to endoglycosidase H (EndoH) treatment. LIMP-2, an endogenously glycosylated protein, served as control. (**E**) Sperm and testis lysates of wild-type (Wt) or C11orf94-deficient (C11 KO) mice were analyzed by Western blotting. (**F**) Expression of C11orf94 within the testis was visualized by IHC using a C11orf94-targeting antibody. Asterisk, unspecific staining. Scale bars, 25 μm. (**G**) Expression of C11orf94 in different spermatid stages was evaluated by staining of testis cryosections from Wt mice with anti-C11orf94 and PNA-FITC as acrosomal marker. Scale bars, 5 μm.

On the basis of the strong down-regulation of C11orf94 in the absence of SPPL2c (*20*), we speculated that both proteins might functionally interact. To understand their potential interplay, we first performed a basic characterization of the biochemical properties of the small 99–amino acid protein based on an expression construct encoding murine C11orf94 equipped with a C-terminal triple-FLAG tag (Fig. 1A). Upon overexpression in HeLa cells, C11orf94-3xFLAG was detected in a tubular network positive for the ER marker protein calnexin by indirect immunofluorescence (Fig. 1B). By contrast, little or no colocalization was observed with the ER-Golgi intermediate compartment marker protein ERGIC-53 or the Golgi marker protein GM130, respectively (Fig. 1B). In agreement with the initial detection of C11orf94 in testis membrane fractions (*20*), TMD prediction using the TMHMM server (v2.0) suggested a membrane-spanning segment between amino acids 9 and 29 at the N terminus of C11orf94, which, however, was assigned as potential signal sequence. By contrast, overexpressed C11orf94-3xFLAG could exclusively be detected in carbonate-washed membrane fractions of transfected HEK293T cells with no detectable levels in the cytosolic fraction (Fig. 1C). This strongly argues for the existence of a functional TMD rather than a signal peptide at the N terminus of C11orf94. To further delineate the topology of the newly found C11orf94 TMD, we fused an artificial N-glycosylation site (sequence NIS) either to the N or the C terminus of C11orf94, which can only be used if the respective part of the mutated protein at least temporarily faces the ER lumen. While C11orf94 does not contain an endogenous N-glycosylation site and therefore was not found to be glycosylated upon overexpression, addition of the NIS motif to the C but not the N terminus of C11orf94 resulted in a clear mass shift of the protein, which was completely reversible by treatment with endoglycosidase H (Fig. 1D). This finding suggests a type II topology of C11orf94, which is remarkable since this orientation is a prerequisite for cleavability of potential substrate TMDs by SPP/SPPL proteases. However, mass spectrometric data from previous studies indicated a destabilization of C11orf94 in the absence of SPPL2c not fitting into a classical protease-substrate relationship.

Since, so far, our analysis of the molecular properties of C11orf94 had exclusively focused on the overexpressed protein, we next characterized expression of C11orf94 in vivo using wild-type and C11orf94-deficient mice, which were obtained from the Mutant Mouse Resource and Research Center at University of California Davis, CA. In these mice, the complete C11orf94 protein coding region was replaced by a β-galactosidase reporter cassette within the C11orf94-encoding *1700029I15Rik* gene (fig. S1A). Success of the targeting strategy was validated by genotyping polymerase chain reactions (PCRs) for both the wild-type and the mutated allele (fig. S1B). Because of the lack of commercial antibodies targeting murine C11orf94, we generated a polyclonal antibody directed against the C terminus of the protein. This antibody specifically detected overexpressed murine and human C11orf94 in HEK cells (fig. S1C). Using this tool, we analyzed endogenous expression of C11orf94 in all major murine organs by Western blotting. While C11orf94 could not be detected in most organs including spleen, lung, brain, bone marrow, liver, and heart (fig. S1, D to I), testis homogenates revealed substantial amounts of the C11orf94 protein (Fig. 1E). By contrast and comparable to SPPL2c, the protein was completely absent from epididymal spermatozoa (Fig. 1E) and the female reproductive tract (fig. S1, J and K). Within the testis, C11orf94 was exclusively detected in wild-type round spermatids as judged from general morphology and nuclear appearance of the expressing cells. Despite some unspecific staining in the tubular lumen, C11orf94 was not identified in any later developmental stage of male germ cells by immunohistochemistry (IHC; Fig. 1F). Because maturation of round spermatids involves several morphologically distinct stages that are associated with extensive rearrangement of acrosomal compartments, we aimed to delineate the exact stage of expression of C11orf94 by costaining with the acrosomal marker peanut agglutinin (PNA)–fluorescein isothiocyanate (FITC) as described in detail in (*25*). Following this approach, applying confocal imaging C11orf94 could only be detected in round spermatids belonging to stages V to VII/VIII with no visible expression in earlier or later stages (Fig. 1G). In these cells, C11orf94 was predominantly localized in a perinuclear but non-acrosomal structure as judged from missing colocalization with PNA-FITC arguing for an ER expression of this protein. These findings strongly suggest a highly specialized and most likely transient function of C11orf94 within the male reproductive tract.

### C11orf94 binds to the catalytic center of SPPL2c

**Fig. 2.**
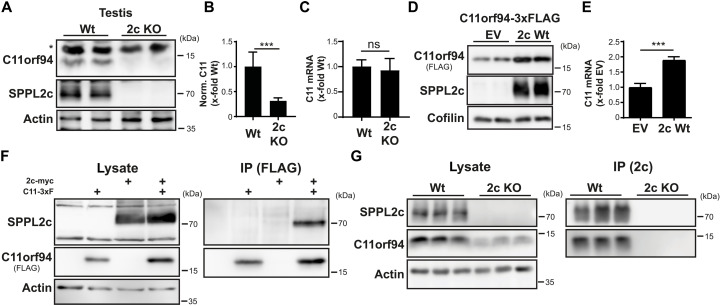
C11orf94 physically interacts with SPPL2c. (**A**) Protein levels of C11orf94 in the testis of either Wt or SPPL2c-deficient (2c KO) mice were determined by Western blotting. The asterisk highlights an unspecific band. (**B**) Quantification of (A). *N* = 2, *n* = 6, two-tailed unpaired Student’s *t* test. (**C**) Expression of the C11orf94 mRNA was analyzed by quantitative PCR using testis cDNA from Wt or *Sppl2c*^−/−^ mice. *N* = 1, *n* = 7 (Wt)/6 (2c KO), two-tailed unpaired Student’s *t* test. (**D**) HEK cells were transiently transfected with C11orf94-3xFLAG alone or together with Sppl2c-myc. Protein levels were visualized by Western blotting. (**E**) Quantification of (D). *N* = 4, *n* = 8, two-tailed unpaired Student’s *t* test. (**F**) HEK cells were transfected with the indicated constructs. After lysis with 1% Triton X-100, pulldown of C11orf94 from the respective lysates was performed using anti-FLAG and protein G agarose beads. Lysates and bead eluates were subjected to Western blot analysis using the indicated antibodies. (**G**) Testes from wild-type or *Sppl2c*^−/−^ mice were lysed in 0.5% CHAPSO. SPPL2c was precipitated from the lysates using a specific antibody and protein G agarose. Lysates and bead eluates were analyzed by Western blotting. ns, not significant. ***P ≤ 0.001.

We now aimed to analyze the potential interplay of C11orf94 with SPPL2c. Since previous mass spectrometric analysis of testis membranes from either wild-type or SPPL2c-deficient mice demonstrated a significant down-regulation of C11orf94 protein levels in the absence of the protease (*20*), we aimed to validate this finding using our novel C11orf94-specific antibody. Western blotting confirmed a reduction of C11orf94 protein levels of approximately 70% in the testis of SPPL2c-deficient mice if compared to wild-type controls (Fig. 2, A and B). By contrast, C11orf94 mRNA levels were not significantly affected upon loss of SPPL2c (Fig. 2C), clearly pointing to a posttranslational stabilization of the C11orf94 protein in a SPPL2c-dependent manner. This was further substantiated by the notion that C11orf94 was significantly enriched by coexpression of SPPL2c in HEK cells (Fig. 2D,E). This is remarkable since, despite fulfilling all known criteria for being a SPPL2c substrate, C11orf94 was not degraded by this protease but stabilized instead.

The stabilization of C11orf94 by SPPL2c raised the question of how this could be achieved. We speculated that physical interaction of both proteins provides the basis for this effect. The intramembrane protease could be efficiently copurified with immunoprecipitated C11orf94 in transfected HEK cells (Fig. 2F). This interaction was highly specific since C11orf94 did not interact with the closely related and also ER-resident SPP intramembrane protease (fig. S2A). Endogenous interaction of SPPL2c and C11orf94 could also be verified in testis homogenates of wild-type mice (Fig. 2G), highlighting the in vivo relevance of the observed protein-protein interaction.

**Fig. 3.**
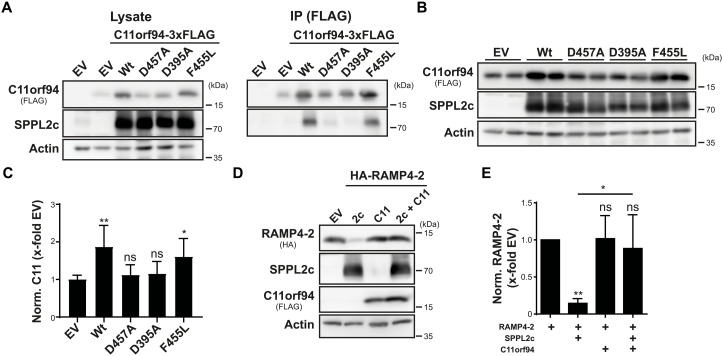
C11orf94 is stabilized by binding to the catalytic center of SPPL2c. (**A**) HEK cells were transiently transfected with the indicated constructs, and C11orf94 was pulled down from the lysates using anti-FLAG and protein G agarose beads. Protein levels in lysates and immunoprecipitated (IP) samples were subjected to Western blot analysis. (**B**) After transfection of HEK cells with the indicated constructs, C11orf94 levels were compared between the samples by Western blot analysis. (**C**) Quantification of (B). *N* = 4, *n* = 8, one-way analysis of variance (ANOVA) with Tukey’s post hoc test. Throughout the figure, statistical indications above the individual bars depict significance against the empty vector (EV)–cotransfected control. (**D**) Processing of RAMP4-2 by SPPL2c (2c) in the presence or absence of C11orf94 (C11) was evaluated by Western blotting of lysates prepared from transiently transfected HEK cells. (**E**) Quantification of (D). *N* = 4, *n* = 4, one-way ANOVA with Tukey’s post hoc test. In addition to significance against the EV-cotransfected sample indicated above the individual bars, statistical comparisons of other samples are indicated with lines. *P ≤ 0.05 and **P ≤ 0.01.

Next, we aimed to analyze how binding to SPPL2c facilitates stabilization of C11orf94. On the basis of the type II orientation of the C11orf94 TMD, we speculated that, comparable to a substrate molecule, C11orf94 is recruited to the catalytic center of SPPL2c where it is bound in a tight manner, thereby escaping so far unspecified degradative pathways. To test this hypothesis, we analyzed binding of C11orf94 to SPPL2c variants carrying mutations in the catalytically relevant aspartate residues (D395A and D457A), which lack proteolytic activity as judged by failure of these mutants to process the established SPPL2c substrate Ribosome-associated membrane protein (RAMP4-2) (fig. S2, B and C). As additional control, SPPL2c F455L harboring a mutation in close proximity to D457A but still being able to proteolyze RAMP4-2 (fig. S2, B and C) was included. As shown in Fig. 3A, mutation of any of the two catalytic aspartates significantly reduced binding of SPPL2c to C11orf94, while introduction of the F455L mutant did not affect the C11orf94-SPPL2c interaction. In line with this, SPPL2c D395A and D457A failed to significantly stabilize C11orf94 upon coexpression in HEK cells, while the neighboring F455L mutation did not affect SPPL2c-mediated stabilization of C11orf94 (Fig. 3, B and C). Binding seemed to directly depend on the catalytic aspartates but not on proteolytic activity in general since mutation of the PALL motif in TMD 9 of SPPL2c, which completely blocked processing of RAMP4-2 (fig. S2, D and E), had no significant effect on the stabilization of C11orf94 (fig. S2, F and G). Since, as a consequence of binding to the catalytic center, C11orf94 would block access of substrate molecules to the active site of SPPL2c, we analyzed cleavage of RAMP4-2 by the intramembrane protease in the presence of C11orf94 by coexpression of these proteins in HEK cells. While RAMP4-2 was efficiently proteolyzed by SPPL2c in the absence of C11orf94, introduction of the SPPL2c interactor almost completely prevented RAMP4-2 processing (Fig. 3, D and E). Similar effects were obtained for other established SPPL2c substrates including Heme oxygenase-1 (HO-1) (fig. S2, H and I), Stx8 (fig. S2, J and K), and PLN (fig. S2, L and M). Again, inhibition was highly specific for SPPL2c since proteolysis of RAMP4-2 by SPP was not affected by the presence of C11orf94 (fig. S2, N and O). This, at least under the used conditions, establishes C11orf94 as the first protein inhibitor of an intramembrane protease directly accessing the catalytic center of an I-CLiP.

### C11orf94 deficiency leads to normozoospermic infertility

**Fig. 4.**
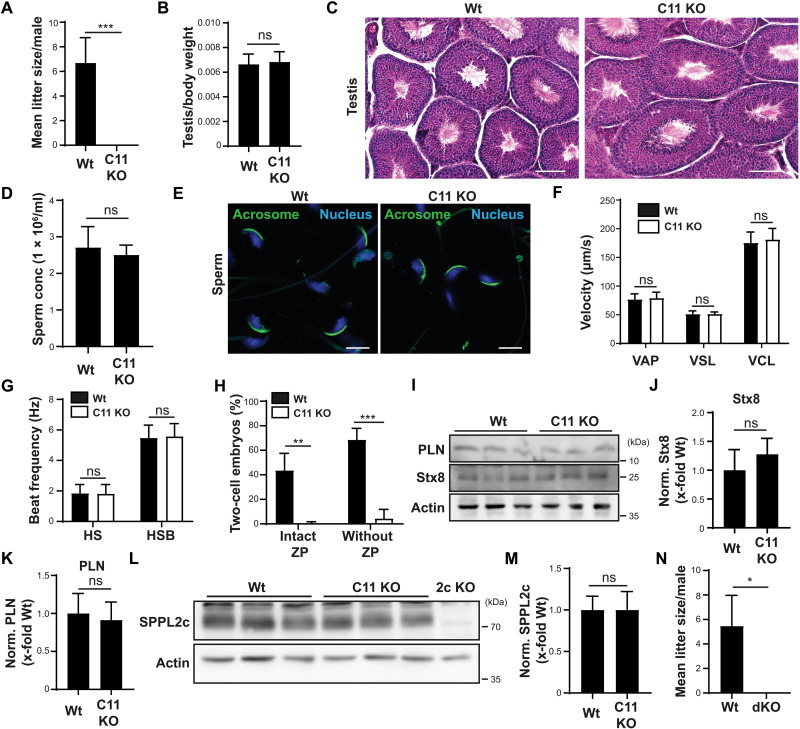
C11orf94 deficiency causes normozoospermic infertility independent of SPPL2c activity. (**A**) Male Wt or C11orf94-deficient (C11 KO) mice were bred with Wt females and mean litter sizes were recorded. *n* = 3 (Wt)/5 (C11 KO). (**B**) Analysis of normalized testis weight. *n* = 10. (**C**) Testis cryosections were subjected to H&E staining. Scale bars, 25 μm. (**D**) Analysis of sperm counts of Wt and C11 KO mice. *n* = 7. (**E**) Epididymal spermatozoa were fixed, and nuclei were visualized with 4′,6-diamidino-2-phenylindole (DAPI), while acrosomes were stained with PNA-FITC. Scale bars, 5 μm. (**F**) Motility parameters of sperm cells were measured by CASA. VAP, average path velocity; VSL, straight line velocity; VCL, curvilinear velocity. *n* = 3. (**G**) Tail beat frequency of epididymal spermatozoa was analyzed upon addition of either HS buffer or HS supplemented with HCO_3_^−^ (HSB). *N* = 3, *n* = 19 sperm per conditions. (**H**) Fertilization capacity of sperm cells from either Wt or C11 KO mice incubated with Wt oocytes with or without ZP was analyzed by IVF. *N* = 2, *n* = 3. (**I**) Testis protein levels of PLN and Stx8 were detected by immunoblotting. (**J**) Quantification of Stx8 levels from (I). *N* = 3, *n* = 9. (**K**) Quantification of PLN levels from (I). *N* = 2, *n* = 6. (**L**) SPPL2c protein levels were analyzed in homogenates from Wt or C11 KO testes by Western blotting. (**M**) Quantification from (L). *n* = 9. (**N**) Wt or C11orf94/SPPL2c double-deficient (dKO) males were bred with Wt females, and mean litter sizes were recorded. *n* = 3. In all cases, unpaired two-tailed Student’s *t* tests were performed. *P ≤ 0.05, **P ≤ 0.01, and ***P ≤ 0.001.

Having identified C11orf94 as potential SPPL2c-inhibiting molecule, we aimed to characterize the physiological role of the C11orf94-SPPL2c interaction by analyzing C11orf94-deficient male mice. Because of the testis-specific expression of C11orf94, we first analyzed the reproductive capacity of these animals. We either crossed male wild-type or *C11orf94^−/−^* mice with wild-type females and recorded litter sizes over a period of at least 3 months per breeding pair. While wild-type males reproduced efficiently during this period, loss of C11orf94 resulted in complete infertility of male mice (Fig. 4A). Under the same breeding regime, SPPL2c-deficient males produce litters of comparable size to wild-type controls, meaning that loss of C11orf94 provokes a much stronger phenotype than loss of the interacting intramembrane protease (*20*).

To obtain insights into the cause of male sterility in *C11orf94^−/−^* mice, we analyzed the male reproductive tract in more detail. C11orf94-deficient males neither had significant differences in testis/body weight ratios (Fig. 4B) nor testicular morphology in general, as judged from hematoxylin and eosin (H&E) staining of testis cryosections (Fig. 4C) if compared to wild-type controls. Sperm cells could be recovered from epididymides of these animals in comparable numbers as from wild-type controls (Fig. 4D). Despite the complete loss of fertility in *C11orf94^−/−^* mice, C11orf94 deficiency did not cause any obvious morphological sperm defects as judged from normally formed sperm heads and intact acrosomes as analyzed by PNA-FITC staining of fixed spermatozoa (Fig. 4E). Since SPPL2c-deficient mice show an about 30% reduction in sperm motility (*20*), we monitored whether this could also be observed in *C11orf94^−/−^* mice. In contrast to loss of SPPL2c, ablation of C11orf94 did not result in any alteration in basic sperm motility parameters (Fig. 4F). In addition, activation of sperm cells was not affected in the absence of C11orf94, as judged from measurement of beat frequency of sperm tails upon stimulation with HEPES-saline (HS) buffer supplemented with bicarbonate (HSB) (Fig. 4G), thereby ruling out motility-related pathways as cause for male infertility in *C11orf94^−/−^* mice. Since, so far, these experiments did not reveal the mechanism underlying normozoospermic infertility in C11orf94-deficient males, we performed in vitro fertilization (IVF) studies using wild-type oocytes and sperm cells from either wild-type or *C11orf94*^−/−^ mice. Sperm from C11orf94-deficient mice completely failed to fertilize oocytes under these conditions (Fig. 4H), although they efficiently bound to the oocytes. The same effect could be observed when the ZP was removed from the oocytes before incubation with the sperm, excluding problems in ZP penetration as underlying mechanism (Fig. 4H). One hour after the beginning of the coincubation, sperm from *C11orf94*^−/−^ mice was efficiently attached to oocytes, suggesting that the observed defects were not due to impaired binding (fig. S3). These findings strongly suggest that a failure of sperm from C11orf94-deficient mice to fuse with oocytes, which is remarkable since, as mentioned above, C11orf94 is not expressed in mature spermatozoa and therefore cannot be involved in sperm oocyte fusion directly.

### The phenotype of C11orf94-deficient mice is independent of the proteolytic function of SPPL2c

As described above, coexpression with C11orf94 leads to the almost complete inhibition of SPPL2c activity in overexpression systems (Fig 3, D and E). At the same time, this binding event is crucial for the stabilization of C11orf94. Therefore, we were interested in which function of the C11orf94/SPPL2c complex might be more relevant for regulation of male fertility: fine-tuning of SPPL2c-mediated intramembrane proteolysis or stabilization of C11orf94 for fulfilling a yet unknown additional function. To address this question, we first assessed protein levels of the established SPPL2c substrates Stx8 and PLN in the testis of wild-type and C11orf94-deficient mice. If loss of C11orf94 would cause hyperactivity of SPPL2c, then both Stx8 and PLN protein amounts should be clearly decreased. Unexpectedly, levels of neither Stx8 nor PLN showed a significant reduction upon loss of C11orf94 (Fig. 4, I to K). This was not caused by a potential compensatory down-regulation of SPPL2c since *C11orf94^−/−^* males displayed comparable levels of the protease in testis homogenates (Fig. 4, L and M), strongly suggesting that the phenotype of C11orf94 deficiency was not related to alterations in proteolytic activity of SPPL2c. To validate this hypothesis, we generated C11orf94/SPPL2c double-deficient mice by crossing animals from both strains. As mentioned above, while C11orf94 deficiency leads to a complete loss of male fertility, *Sppl2c^−/−^* males still produce normal-sized litters when bred with wild-type females. If the loss of fertility of *C11orf94^−/−^* males would depend on increased SPPL2c activity, it should therefore be compensated by the simultaneous depletion of the protease. By contrast, as depicted in Fig. 4N, *C11orf94^−/−^*/*Sppl2c^−/−^* comparable to C11orf94 single-deficient males completely failed to generate litters under the used breeding regime.

### C11orf94 regulates the ER export of Izumo1

Since these findings clearly suggested that the normozoospermic infertility of *C11orf94*^−/−^ males is not related to altered activity of SPPL2c, we analyzed the C11orf94 interactome using an unbiased immunoprecipitation (IP)–MS approach to find alternative pathways in which C11orf94 might be involved. For this purpose, we coupled the newly generated C11orf94-targeting antibody to Sepharose beads and precipitated C11orf94 from ([3-Cholamidopropyl]dimethylammonio)-2-hydroxy-1-propansulfonat (CHAPSO) homogenates of wild-type testes. C11orf94-deficient testis homogenates served as negative control. Precipitated proteins were digested by trypsin directly on the beads and subjected to mass spectrometric analysis. Under these conditions, the established C11orf94 interactor SPPL2c could be readily precipitated (Table 1). The only other protein that was found to be enriched in at least two of the three repetitions in wild-type precipitates was Izumo1. Izumo1 is a type I transmembrane protein that can be found in testis and mature sperm and is essential for sperm-oocyte fusion (*4*). Upon induction of the acrosome reaction, Izumo1 is exposed at the sperm surface where it can interact with its oocyte counterpart Juno to initiate gamete fusion (*26*). Deficiency of Izumo1 and several other potential interaction factors leads to normozoospermic infertility as observed for *C11orf94^−/−^* males (*4*).

**Table 1.** List of proteins enriched in Wt precipitates in AP/MS experiments. Wt/C11orf94 knockout fold changes (rounded) are depicted. All proteins with at least twofold higher abundance in Wt samples that were identified in at least two independent experiments were considered. n.d., not detected. OS, Organism name; OX, Organism Identifier; GN, Gene Name; PE, Protein existence; SV, Sequence version.

**Description**	**Accession**	**Fold enrichment**		
		**Run 1**	**Run 2**	**Run 3**
Uncharacterized protein C11orf94 homolog OS = *Mus musculus* OX = 10090 PE = 3 SV = 1	Q8CF31	54	4.5	92
Signal peptide peptidase-like 2C OS = *M. musculus* OX = 10090 GN=Sppl2c PE = 2 SV = 1	A2A6C4	6.6	12	14
Izumo sperm-egg fusion protein 1 OS = *M. musculus* OX = 10090 GN=Izumo1 PE = 1 SV = 1	Q9D9J7	2.4	n.d.	9.7

**Fig. 5.**
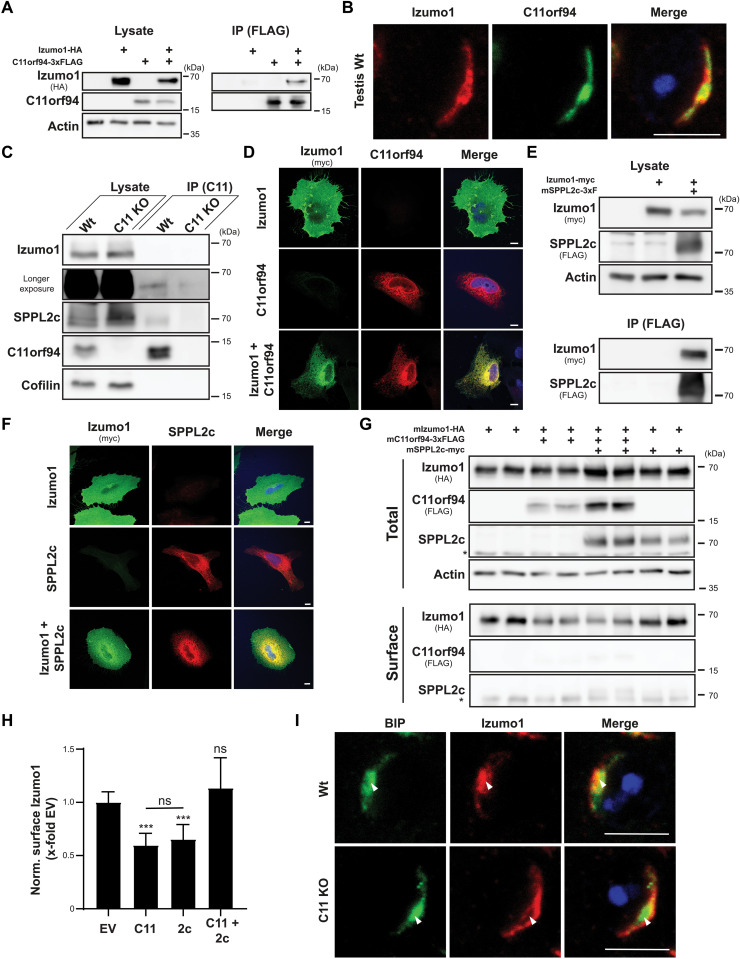
C11orf94 regulates ER exit of Izumo1. (**A**) CHAPSO-solubilized proteins from transfected HeLa cells were precipitated using anti-FLAG beads. Total lysates and bead eluates (IP) were analyzed by Western blotting. (**B**) Testis cryosections from Wt mice were stained with anti-Izumo1, anti-C11orf94, and DAPI. Scale bar, 5 μm. (**C**) C11orf94 was precipitated from CHAPSO homogenates from Wt or *C11orf94*^−/−^ (C11 KO) testes using anti-C11orf94 Sepharose. Lysates and eluates were analyzed by Western blotting. (**D**) Transfected HeLa cells were fixed and subjected to indirect immunofluorescence analysis. Scale bars, 10 μm. (**E**) Interaction of Izumo1-myc and SPPL2c-3xFLAG was analyzed as described in (A). (**F**) Subcellular localization of SPPL2c-3xFLAG and Izumo1-myc was visualized by indirect immunofluorescence in transfected HeLa cells. Scale bars, 10 μm. (**G**) After biotinylation of surface proteins, transfected HeLa cells were lysed and biotinylated proteins were precipitated using streptavidin-coated beads. Lysates and bead eluates (IP) were subjected to Western blot analysis. Asterisk, unspecific band. (**H**) Quantification of (G). *N* = 3 to 6, *n* = 6 to 12, one-way ANOVA with Tukey’s post hoc test. Statistical significance compared to EV-transfected control is indicated above the bars. (**I**) Colocalization of Izumo1 with the ER marker protein BIP in Wt or C11 KO round spermatids was analyzed by IHC. ***P ≤ 0.001. Scale bars, 5μm.

We aimed to validate the C11orf94-Izumo1 interaction suggested by the MS data. As shown in Fig. 5A, Izumo1–hemagglutinin (HA)-tagged Izumo1 could be coprecipitated with C11orf94 from transiently transfected HeLa cells. In the testis, Izumo1 partially colocalized with C11orf94 in round spermatids, which therefore represent the relevant cell type for interaction of both proteins (Fig. 5B). In line with these findings and the initial mass spectrometric analysis, Izumo1 was also specifically coprecipitated with C11orf94 from testis homogenates, although at low levels and some unspecific binding of Izumo1 to the beads used for precipitation (Fig. 5C). However, the limited efficiency in this setup might also be partially caused by the potential overlap of the Izumo1 binding site with the epitope of the C11orf94-targeting antibody.

Having successfully validated the C11orf94-Izumo1 interaction, we went on to analyze its functional relevance. Since Izumo1 resides at the plasma membrane of transfected cells (*13*) while C11orf94 resides in ER membranes (Fig. 1A), we first were interested in where both proteins might interact. When expressed alone, Izumo1 was predominantly detected at the plasma membrane of transfected HeLa cells (Fig. 5D). Unexpectedly, upon coexpression of C11orf94, localization of Izumo1 largely shifted to the ER where it significantly colocalized with the small ER protein (Fig. 5D). This effect was highly specific since C11orf94 did influence neither localization of the unrelated type I–oriented control protein cell adhesion molecule 1 (fig. S4A) nor ER export and subsequent γ-secretase–dependent processing of the trafficking reporter NotchΔE–enhanced green fluorescent protein (eGFP) (fig. S4B). In addition, SPPL2c was able to physically interact with Izumo1 in the used overexpression system (Fig. 5E). However, despite minor colocalization within the ER, interaction of both proteins did not result in a clear ER retention of Izumo1 comparable to the situation observed for C11orf94, as judged by indirect immunofluorescence (Fig. 5F). These findings were substantiated by the analysis of surface Izumo1 levels by surface biotinylation experiments. While coexpression of C11orf94 caused an almost 50% decline of surface Izumo1 levels, cotransfection of SPPL2c with Izumo1 did not affect Izumo1 transport (Fig. 5, G and H). Furthermore, expression of C11orf94 and SPPL2c together with Izumo1 did not recover surface Izumo1 levels, meaning that SPPL2c did not act as a release factor for Izumo1 (Fig. 5, G and H). By contrast, coexpression of SPPL2c increased interaction of Izumo1 and C11orf94 (fig. S4C), suggesting that SPPL2c acts as a scaffold protein supporting C11orf94 in Izumo1 recruitment. To validate that ER retention of Izumo1 by C11orf94 also takes place in vivo, we analyzed ER levels of Izumo1 in round spermatids by IHC using the chaperone Binding immunoglobulin protein (BIP) as ER marker protein (Fig. 5I). Similar to C11orf94, expression of BIP in round spermatids concentrated in a condensed organelle close to the nucleus with only minor levels in a cap-like structure. While in wild-type round spermatids, a significant fraction of Izumo1 could be observed in these condensed structures, depletion of C11orf94 led to a reduction of colocalization of Izumo1 and BIP specifically in this compartment. This suggests that C11orf94 acts as trafficking brake for the ER export of Izumo1 not only in cell-based overexpression systems but also in vivo.

### C11orf94 and SPPL2c coordinate Izumo1 complex assembly

Having validated the C11orf94-Izumo1 interaction and its functional consequences, we went on to analyze its physiological relevance with regard to the phenotype of *C11orf94*^−/−^ mice by characterizing stability and trafficking of Izumo1 in later stages of germ cell development. Izumo1 localization within acrosomal structures of elongated spermatids (fig. S5A) and protein levels in total testis (fig. S5, B and C) were unaltered in C11orf94-deficient mice if compared to wild-type controls. In agreement, no significant alterations in Izumo1 protein amounts could be detected in epididymal spermatozoa both before and after calcimycin-induced acrosome reaction (fig. S5, D to G). This was in line with unaltered subcellular localization of Izumo1 both in noncapacitated (fig. S5H) and acrosome-reacted cells (fig. S5I), in which Izumo1 could be detected predominantly spread throughout the whole sperm head with only a minor fraction of cells revealing relocalization of Izumo1 limited to the equatorial segment (fig. S5J). These findings suggest a rather unaltered expression and localization of Izumo1 in mature spermatozoa in the absence of C11orf94, excluding these processes as reasons for the infertility of C11orf94-deficient male mice.

**Fig. 6.**
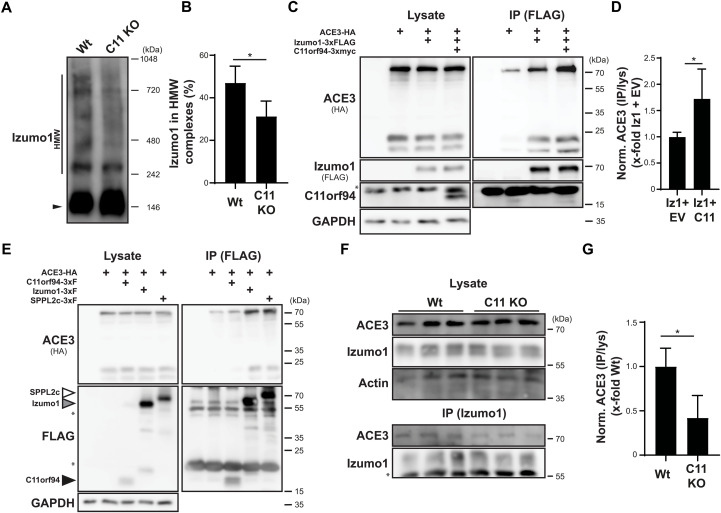
C11orf94 regulates formation of Izumo1 complexes. (**A**) Analysis of Izumo1-containing complexes in the testis of Wt or C11orf94-deficient (C11 KO) mice. Homogenized testes were lysed and subjected to blue native PAGE with subsequent Western blotting. Monomeric (filled arrow head) Izumo1 and Izumo1 in HMW complexes (HMW) are highlighted. (**B**) Quantification of (A). *N* = 3, *n* = 5, two-tailed unpaired Student’s *t* test. (**C**) HEK293T cells were transfected with ACE3-HA alone or in combination with Izumo1-3xFLAG and C11orf94-3xmyc. Izumo1-3xFLAG was precipitated from CHAPSO homogenates of these cells using FLAG-specific beads, and proteins were detected in whole-cell lysates (lysate) or immunoprecipitated (IP) samples. Asterisk, antibody band. (**D**) Quantification of (C). *N* = 2, *n* = 5, two-tailed unpaired Student’s *t* test. (**E**) Interaction of ACE3-HA with C11orf94-3xFLAG, SPPL2c-3xFLAG, or Izumo1-3xFLAG was analyzed by coimmunoprecipitation experiments using CHAPSO lysates of transfected HEK293T cells and anti-FLAG beads. Asterisk, antibody band. (**F**) Izumo1 was precipitated from CHAPSO lysates of noncapacitated sperm cells from either Wt or C11 KO mice, and lysates and IP samples were analyzed by Western blotting. Actin served as loading control. Asterisk, antibody band. (**G**) Quantification of (F). *N* = 1, *n* = 3, unpaired Student’s *t* test. *P ≤ 0.05.

**Fig. 7.**
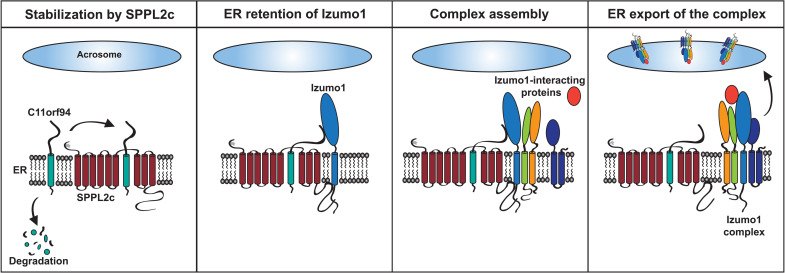
Proposed model for the function of C11orf94 in the testis. Within round spermatids, C11orf94 binds to the catalytic center of SPPL2c, which protects it from degradation by a yet unknown mechanism. Izumo1 is recruited to the C11orf94-SPPL2c complex, which results in retention of the fusion factor within the ER. The C11orf94/SPPL2c/Izumo1 complex serves as platform for recruitment of several Izumo1-interacting proteins including ACE3 but potentially also other fusion-relevant molecules. Last, interaction with these proteins leads to ER export of Izumo1 complexes.

On the basis of reports that Izumo1 forms large complexes that also seem to involve other proteins (*14*) and the recent discovery of several Izumo1-interacting proteins that affect gamete fusion (*13*, *16*, *17*, *27*), we speculated that the transient retention of Izumo1 within the ER might be required for efficient assembly of Izumo1-containing complexes. To monitor complex formation, we analyzed Izumo1 complex formation by blue native PAGE (polyacrylamide gel electrophoresis) of testis homogenates with subsequent Western blotting. As depicted in Fig. 6A and quantified in Fig. 6B, incorporation of Izumo1 in HMW complexes was significantly reduced in testes from C11orf94-deficient mice. At the same time, we observed increased amounts of monomeric Izumo1, indicating that, in line with previous findings (fig. S5, B to G), the reduced Izumo1 levels in HMW complexes were not caused by a general decrease in the Izumo1 protein quantity in the testis. To further delineate whether reduced Izumo1 complex formation resulted from its decreased interaction with potential interaction partners, we decided to use angiotensin-converting enzyme 3 (ACE3) as model protein. Although a disturbed interaction of this molecule with Izumo1 is most likely not responsible for the phenotype of C11orf94-deficient mice since loss of ACE3 does not affect male fertility (*28*), together with GLIPR1L1 (*16*), ACE3 remains the, so far, only in vivo validated interaction partner of Izumo1 (*28*). Furthermore, an antibody reliably detecting the protein at the endogenous level is commercially available. We tried to overcome the lack of antibodies targeting the more fusion-relevant potential Izumo1 interactors SOF1 and SPACA6 and tested several custom-made antibodies and a commercial antibody designed to target these molecules. However, none of these detected SOF1 or SPACA6 at the endogenous level in sperm or testis lysates in our assay conditions, excluding these proteins from further analysis (fig. S6, A and B). Since the SOF1 targeting antibody described in (*13*) has been used before successfully in Western blot applications, this was most likely caused by differences in the respective used lysis protocols. For these reasons, we cloned murine ACE3 equipped with a C-terminal HA-tag from testis cDNA and analyzed its coprecipitation with Izumo1 in dependence of the presence of C11orf94 in transiently transfected HEK239T cells. As expected, ACE3 could be efficiently coprecipitated with Izumo1 even in the absence of C11orf94 (Fig. 6C). However, the interaction was significantly increased to about 1.7-fold in the presence of C11orf94-3xmyc (Fig. 6, C and D). This was not caused by direct interaction of ACE3 with C11orf94, as we failed to enrich ACE3 upon IP of coexpressed C11orf94-3xFLAG above the level of background binding to the beads (Fig. 6E). By contrast, ACE3 could be efficiently coprecipitated with SPPL2c-3xFLAG under the same experimental conditions. These findings are in line with our hypothesis that C11orf94 could act as a gatekeeper for Izumo1 complexes by retaining this protein in the ER, while SPPL2c rather represents a non-essential scaffold molecule. Having demonstrated in a cell-based model that C11orf94 is capable of increasing the interaction of Izumo1 with its established interaction partner ACE3, we aimed to validate this finding in vivo. To analyze whether the observed reduction in Izumo1 HMW complexes in the testis of C11orf94-deficient mice is reflected by a diminished interaction of Izumo1 with other proteins and is not exclusively based on reduced homomultimerization (*14*, *29*), we lastly precipitated Izumo1 from noncapacitated epididymal spermatozoa from either wild-type or C11orf94-deficient males and analyzed the precipitates for the presence of ACE3. Although being coprecipitated at low levels, we could copurify about 50% less ACE3 with Izumo1 from sperm of C11orf94-deficient males (Fig. 6, F and G). This strongly supports a critical role of C11orf94 for the organization and assembly of Izumo1 complexes in vivo, which appears to be essential for gamete fusion. In our proposed model, C11orf94 acts as an essential gatekeeper for Izumo1 complex formation by retaining this protein in the ER of round spermatids by physical interaction. Although not being essential for male fertility itself, the intramembrane protease SPPL2c supports the function of C11orf94 in several ways. On the one hand, the protease stabilizes C11orf94 within its catalytic center, thereby preventing its degradation by a yet unknown mechanism. On the other hand, it acts as scaffold molecule by recruiting not only both Izumo1 but also other interaction partners such as ACE3. Last, upon complete assembly of the Izumo1 complex, its ER retention is released, facilitating its export to the acrosome (Fig. 7).

## DISCUSSION

Our findings establish C11orf94 as key player in male fertility, most likely by affecting the formation of Izumo1 complexes. Because of its strong relevance for male fertility, we therefore propose “Frey” as a novel name for C11orf94 based on the Norse god of fertility. In addition, the interaction of Frey with SPPL2c represents a novel mode of intramembrane protease action in which the catalytic center of an active protease does not act as a degrading but rather a protective element for the interacting protein. This is remarkable, since Frey in general fulfills all requirements for being a bona fide substrate of SPPL2c itself and physically interacts with the protease. This largely depends on the two aspartates within the active site of SPPL2c, suggesting that Frey is directly recruited to the catalytic center of the I-CLiP. Although this does not fully exclude binding of Frey to potential exosites on the enzyme required for substrate recruitment, these data are suggestive of a model in which Frey specifically interacts with the catalytic center of SPPL2c with high affinity, thereby blocking access of potential substrate molecules. How this high affinity can be obtained and whether an inhibitory motif within the Frey TMD might contribute to this will be a central question of further research, especially since analysis of cleavage sites of aspartic intramembrane proteases has not revealed any obvious consensus cleavage sites yet (*21*, *23*, *30*).

Stabilization of Frey by SPPL2c represents the first described nonproteolytic function of an SPPL2c protease. Noncatalytic functions have been determined for several other I-CLiPs including SPPL3 (*31*) and γ-secretase (*32*–*35*). In the case of rhomboid proteases, even a whole class of catalytically inactive homologs termed iRhoms exists (*36*). These proteins have been implicated in regulation of several biological pathways by controlling trafficking and activity of the A disintegrin and metalloproteinase 17 (*37*–*39*). The interaction of Frey and SPPL2c does not match any of the described examples since, in this case, a potentially active protease uses residues directly involved in catalysis for exerting a nonproteolytic function. This demonstrates the broad versatility of protease folds for the recognition of TMDs for divergent biological purposes.

Our findings establish Frey as the first protein inhibitor of an aspartic intramembrane protease (Fig. 3). The discovery of a small membrane protein acting as direct inhibitor of an intramembrane protease opens the perspective of the existence of similar proteins modulating the activity of other I-CLiPs. Small protein inhibitors of the *Bacillus subtilis* metallointramembrane protease Stage IV sporulation protein FB (SpoIVFB) have been recently identified that, comparable to Frey, bind to the catalytic center of the intramembrane protease (*40*). By contrast, for mammalian I-CLiPs, only few regulatory proteins have been described. In the case of γ-secretase, several activating proteins were found (*41*–*43*). However, the underlying mechanisms are completely different from that proposed for Frey and SPPL2c. Reanalysis of existent proteomic datasets from protease-deficient cell lines and tissues with a focus on proteins down-regulated in the absence of the protease therefore might represent an important approach to identify potential novel I-CLiP regulators.

While modulation of SPPL2c activity by Frey seems to be of limited physiological relevance, the infertility phenotype of Frey-deficient mice relies on a completely independent function in gamete fusion. On the basis of our findings, we propose a model for Izumo1 complex assembly (Fig. 7). To prevent ER export of incomplete and nonfunctional Izumo1 complexes, Izumo1 is retained in the ER by physical interaction with Frey. This protein is stabilized within the catalytic center of the intramembrane protease SPPL2c which, although not being essential for male fertility itself, serves as scaffold for Izumo1 complex assembly. ER retention of Izumo1 by Frey can be released by incorporation of Izumo1-interacting proteins into the complex.

Our findings suggest that formation of Izumo1 complexes is regulated by an interconnected web of partially transient protein-protein interactions that control protein trafficking. Frey and Izumo1 take over central parts in this process. In agreement with the less pronounced phenotype of SPPL2c-deficient males (*20*), this intramembrane protease rather appears to fulfill a supportive role in this context by acting as non-essential scaffold protein interacting with all complex members analyzed so far. Despite its central position within the found interactive network, loss of SPPL2c does not lead to male infertility, arguing for a potentially redundant function of this protein. While our data suggest that the stabilizing effect of SPPL2c on Frey seems to be highly specific, other membrane molecules might take over the described scaffolding function of SPPL2c under knockout conditions. Whether SPPL2c really plays a redundant role for Izumo1 complex formation and which proteins might take over its function upon loss of the protease remain subject to future research.

Beyond its connection to Frey and Izumo1, SPPL2c also fulfills additional functions within the testis including regulation of sperm motility and early acrosome biogenesis (*20*, *24*). In both cases, this was attributed to proteolytic processing of tail-anchored proteins. Since, at least under overexpression conditions, cleavage of these molecules by SPPL2c was efficiently blocked by Frey (Fig. 3 and fig. S2), this suggests the existence of a subset of SPPL2c molecules that is not directly involved in Frey binding. Therefore, it will be interesting to analyze in detail how SPPL2c affects other sperm fusion proteins and how nonproteolytic and catalytic activities of this protease are balanced in vivo.

Our data identify Frey as previously unrecognized regulator of gamete fusion. Although its involvement in other pathways cannot be excluded and should be investigated in more detail in future studies, our findings suggest that Frey acts as an essential gatekeeper for regulation of Izumo1 trafficking and complex formation in male germ cells. The model that we propose for Izumo1 complex formation presented in Fig. 7 implies that these assemble in early stages of spermatogenesis in the ER. In this step, at least one or several fusion-relevant molecules must be incorporated into the nascent Izumo1 complex in a Frey-dependent manner before its export to the acrosome. While interaction with this or these core partner(s) according to the model needs to be established early and therefore has to remain stable, Izumo1 complexes might be modulated further either during their transport to the acrosome or at later stages during capacitation or acrosome reaction. This modulation might involve both modulation of complex composition and other posttranslational modifications such as phosphorylation that might fine-tune the activity of the complex. As demonstrated for ACE3, interaction partners can be lost after acrosome reaction (*28*), highlighting the, in principle, flexible nature of Izumo1 complexes. Since the precise composition of Izumo1 complexes remains enigmatic, this can only be subject to speculations. A comparative analysis of Izumo1 complexes from round spermatids and mature spermatozoa in an either noncapacitated or acrosome-reacted state might help uncover the fate and composition of Izumo1 complexes during the elaborate steps of spermiogenesis and sperm activation.

Despite the evidence for a function of Frey in the context of Izumo1 complex formation, several open questions remain. As stated before, in our study, we concentrated on the incorporation of ACE3 in Izumo1 complexes mainly because of technical limitations with regard to available antibodies. However, since genetic ablation of ACE3 does not cause a significant reduction in male fertility (*28*), it is unlikely that a reduced interaction of ACE3 with Izumo1 is underlying the infertility of Frey-deficient males. In recent years, several potential interaction partners of Izumo1 have been proposed on the basis of cell-based overexpression systems, which include SOF1, FIMP, SPACA6, and Tmem95 (*13*, *17*, *19*, *27*). Although, so far, none of these interactions have been validated in vivo, loss of any of these proteins leads to normozoospermic infertility, as also observed in Izumo1- and Frey-deficient mice. Therefore, it appears likely that interaction of one or several of these molecules with Izumo1 might be essential for male fertility and that the physical connection between these two molecules might be enabled by the ER retention of Izumo1 by Frey. However, the composition of the HMW complexes in which Izumo1 resides in male germ cells (*14*) is still enigmatic. Therefore, even a so far unidentified interaction partner of Izumo1 might be responsible for the fusion defect in Frey-deficient spermatozoa. In addition to demonstrating the strong necessity to close our gaps in terms of identification of the proteins involved in gamete fusion, our current study emphasizes the additional need to characterize the interplay of these proteins in more detail.

Another question that remains is how ER retention of Izumo1 and especially release of the fully assembled complex are mediated on the molecular level. Typically, ER retention and export are regulated by the presentation or masking of specific ER retention signals within secretory proteins (*44*). In the case of γ-secretase, it has been demonstrated that these motifs can efficiently regulate protein complex assembly (*45*, *46*). By contrast, ER retention of Izumo1 is not mediated by an intrinsic retention signal but is controlled by Frey acting as ER anchor protein. On the basis of the plethora of Izumo1-interacting proteins, which as shown for ACE3 (Fig. 6E) can additionally also interact with the scaffolding intramembrane protease SPPL2c, this might allow a more efficient concentration of sperm fusion proteins within the ER. The molecular details of how the Izumo1-Frey interaction leads to ER retention remain to be elucidated, for example, by extensive mutational analysis of both proteins in cell-based overexpression systems.

The same also applies for the ER export of fully assembled Izumo1 complexes. Again, addressing this question is hampered by our current lack of knowledge about the concrete molecular composition of Izumo1 complexes. In the case of the γ-secretase complex, masking of ER retention signals, for example, by integration of presenilin-interacting molecules such as PEN2 into the nascent complex, allows its ER export (*46*). In the case of Izumo1 and Frey, one possible model would be that the physical interaction of both molecules, which most likely takes place in the ER lumen, is replaced by the interaction of Izumo1 with another interaction partner that occupies the same binding site in the sperm fusion factor. Alternatively, complex assembly might induce a degradative signal initiating Frey degradation, thereby enabling the ER export of the Izumo1 complex. Since both scenarios remain speculative at the moment, they will need to be evaluated in future studies.

The function of Frey in the murine testis has also been subject to another study published as a preprint during the course of the revision of this manuscript (*47*). Using comparable cellular and murine model systems, the authors of this study come to very similar results especially regarding the basic molecular properties of Frey and the infertility phenotype of Frey-deficient male mice. In addition, both studies suggest a strong influence of Frey [that is called 1700029I15Rik in (*47*) according to the name of its murine gene] on the proper formation of acrosomal protein complexes based on affinity purification (AP)/MS analysis of Frey-interacting proteins. However, despite the very obvious similarities in study design and key findings, the proposed mechanisms underlying the function of Frey differ. While we suggest Frey as a direct gatekeeper of Izumo1-trafficking, which is based on transient ER retention of this molecule by physical interaction, the authors of the other study identify several ER-resident chaperones Protein disulfide-isomerase A3 (PDIA3), trafficking regulators [transmembrane emp24 domain 4 and 10 (TMED4 and TMED10)], and proteins involved in N-glycosylation [ribophorin II (RPN2), Oligosaccharyltransferase complex (OSTC), and Defender against cell death 1 (DAD1)] to connect with Frey, which thereby more generally affects posttranslational modification and sorting of acrosomal proteins. In agreement with these findings, loss of Frey is demonstrated to result in increased ER stress in sperm cells. A potential role of Frey in the trafficking of Izumo1 or formation of Izumo1 complexes has not been investigated in the other study most likely since Izumo1 was not identified in the AP/MS approach. On the contrary, we could not identify any of the Frey interactors postulated in the other study to interact with Frey in our MS datasets. A more detailed analysis of the ER stress response pathways in not only round spermatids but also mature spermatozoa will be required to shed light into a potential involvement of Frey in this process. The authors of the other study identify a strong down-regulation of SPACA6 in the sperm of Frey-deficient mice, which might represent an explanation for the infertility of these mice (*13*, *17*, *47*). Whether this strong down-regulation also holds true for other potential Izumo1-interacting molecules such as FIMP and SOF1 and whether it relates to disturbed posttranslational modification of these molecules, reduced interaction with Izumo1, or yet uncharacterized other pathways of Frey activity remain central questions for future research projects.

On the basis of the infertility phenotype of male *Frey*^−/−^ mice suggested by both studies, one could speculate that this might also lead to normozoospermic infertility in humans. However, until today, no such mutations have been identified in the human *FREY* gene. Nevertheless, with regard to the high conservation of Frey across mammalian species, it would be interesting to screen patient cohorts suffering from idiopathic infertility for corresponding mutations. The so far only identified genetic disorder with alterations in the *FREY* locus is a microdeletion on chromosome 11 causing the Potocki-Shaffer syndrome that, among others, is connected to not only the development of seizures and intellectual disability but also morphological abnormalities within the genitourinary tract (*48*). However, no data regarding fertility of male patients with Potocki-Shaffer syndrome are available. In addition, the responsible microdeletion encompasses several other genes, which are more likely to contribute to the strong disease phenotype (*49*, *50*). Furthermore, in Potocki-Shaffer syndrome, only one chromosome is affected, meaning that the associated phenotypes result from haploinsufficiency. Since *Frey*^+/−^ mice reveal no alterations in fertility, it is unlikely that fertility would be affected under these conditions because of alterations in Frey protein levels. The search for mutations in the corresponding gene is further complicated by the fact that, until today, only few data regarding potential mutations causing normozoospermic infertility in humans are available. Even for Izumo1, no human mutations affecting male fertility have been reported yet. This clearly highlights the need for a more detailed analysis of genetic alterations underlying normozoospermic infertility.

In summary, our findings reveal an unexpected mode of intramembrane protease action involved in the regulation of male fertility. We provide a model for the assembly of Izumo1-containing complexes, which will help elucidate molecular pathways essential for murine and, potentially, also human reproduction.

## MATERIALS AND METHODS

### Mouse strains

Breeding of all mouse lines and IVF experiments were approved upon ethical evaluation by the Landesdirektion Sachsen (DD24.1-5131/450/12, DD24.1-5131/450/55, and 25-5131/502/8). SPPL2c-deficient mice have been described in (*20*). Mice deficient for C11orf94 were recovered from frozen spermatozoa obtained from the Mutant Mouse Resource and Research Centers (MMRRC) Repository [C57BL/6N-*1700029I15Rik*^tm1.1(KOMP)Vlcg^]. C11orf94/SPPL2c double-deficient mice were generated by crossing *SPPl2c*^−/−^ mice with *C11orf94*^−/−^ mice. All mouse lines used had a C57BL/6 N*Crl* background. Animal work was conducted in agreement with the Guide for the Care and Use of Laboratory Animals of the German Animal Welfare Act on protection of animals and guidelines approved by the University of Duisburg-Essen Animal Care and Use Committees [Landesamt für Natur, Umwelt und Verbraucherscutz Nordrhein-Westfalen (LANUV), protocol AZ84-2.4.17.A133]. Animal experiments were planned and conducted following the 3R (Replacement, Reduction and Refinement) principles. Mice were genotyped using the following primers: Reg-1700029I15Rik-wtF, TGGAAGAGGCTGACCATTATCC and Reg-1700029I15Rik-wtR, CAGCTCTCAAGAGGCAGTCTGG [expected band size, 304 base pairs (bp)] and Reg-LacF, ACTTGCTTTAAAAAACCTCCCACA and Reg-1700029I15Rik-R, GTAAGGAAATGGCAACGTGTGACC (644 bp).

### In vitro fertilization

Superovulation was induced in 4-week-old C57BL/6 *NCrl* females by injection of 5-IU pregnant mare serum gonadotropin and 5-IU human chorionic gonadotropin 46 or 24 hours before oocyte isolation, respectively. Oocytes were isolated from oviducts, and cumulus cells were removed with by treatment with hyaluronidase (0.5 mg/ml; Sigma-Aldrich). When IVF experiments were performed in the absence of the ZP, cumulus-free oocytes were additionally treated for 20 s with acidic Tyrode’s solution (Sigma-Aldrich). For isolation of sperm, both epididymides isolated from a single male mouse were mechanically disrupted in cryoprotective agent (CPA) buffer (18% raffinose and 3% milk powder in sterile water), and sperms were allowed to swim out for 15 min. After counting, 5 × 10^5^ sperm cells were added to the oocytes in EmbryoMax Human Tubal Fluid (Merck Chemicals GmbH). After 5 hours, oocytes were transferred to M16 medium (Sigma-Aldrich). Binding of sperm cells to the oocytes was monitored 1 hour after insemination. Embryo development was assessed microscopically 24 and 48 hours after fertilization.

### Computer-assisted sperm analysis

For computer-assisted sperm analysis (CASA), epididymal sperms from adult male mice were collected by swim-out procedure from the epididymis in standard HS buffer [135 mM NaCl, 5 mM KCl, 2 mM CaCl_2_, 1 mM MgCl_2_, 20 mM Hepes, 5 mM glucose, 10 mM dl-lactic acid, and 10 mM pyruvic acid (pH 7.4)]. Mouse sperm were washed twice in HS medium (5 min at 300*g*) and resuspended to a final concentration of 1 × 10^6^ cells/ml in HS buffer or HSB buffer, containing 15 mM HCO_3_^−^. To both media, 5% bovine serum albumin (BSA) was added to reduce unspecific binding of sperm head to the glass surface. For analysis, 30 μl of the sperm suspension was loaded into a 100-μm-deep chamber slide (Leja Products B.V.), and sperm motility parameters were determined using the IVOS II CASA System (Hamilton Thorne). The parameters measured were the sperm concentration (cells/ml), average velocity, straight-line velocity, and curvilinear velocity in micrometers per second.

### Beat frequency analysis

Flagellar beat frequency was determined as described previously (*51*, *52*). In short, beat frequency was measured using an inverted microscope (Nikon Diaphot 300) with a 40× 0.65–numerical aperture objective and a MotionScope M3-mono fast speed camera (Imaging Solutions), recorded at 300 Hz. Images were collected using the MotionStudio 64 software (Imaging Solutions) and stored in TIFF format. Determination of single-sperm beat frequency was performed by a semiautomated analysis software written in Igor Pro (WaveMetrics) (*51*).

### Sperm isolation

Epididymides of a single male mouse were excised and opened up in 3 ml of noncapacitating Toyoda-Yokoyama-Hosi (TYH) medium (119.37 mM NaCl, 4.78 mM KCl, 1.19 mM KH_2_PO_4_, 1.19 mM MgSO_4_, 5.56 mM glucose, 1.71 mM CaCl_2_, and 0.5 mM Na-pyruvate), and sperms were allowed to swim out for 30 min at 37°C. Afterward, the suspension was filtered through a 100-μm cell strainer (Greiner), the total volume was adjusted to 4 ml, and the cells were counted using M-NZ Disposable Hemocytometer chips (Kisker Biotech). Afterward, cells were spun down for 5 min at 1000*g* for 2 min and processed further for Western blot analysis or indirect immunofluorescence. Alternatively, cells were capacitated using 25 mM NaHCO_3_ (Roth) and BSA (5 mg/ml; Roth) for 45 min at 32°C, followed by induction of the acrosome reaction for an additional 45 min using 10 μM calcimycin (Sigma-Aldrich).

### Plasmids

Open reading frames encoding C11orf94, Izumo1, SOF1, SPACA6, and ACE3 were amplified by PCR from murine testis cDNA and equipped with C-terminal 3xFLAG, (3x)myc, or HA tags, respectively. Coding sequences were inserted into the pcDNA3.1 hygro^+^ vector (Thermo Fisher Scientific) using Hind III and Xho I (C11orf94, SPACA6, and Izumo1) or Bam HI and Xho I (SOF1 and ACE3) restriction sites. For analysis of the membrane topology of C11orf94, an artificial NIS motif was added to the N or C terminus of the protein, respectively. Plasmids encoding NotchΔE-eGFP (*53*), mSPPL2c-myc, and mSPP-myc and the catalytically inactive mSPPL2c D457A variant (*20*) have been described previously. The mutations D395A and F455L were introduced into the SPPL2c-coding sequence by site-directed mutagenesis, and the resulting open reading frames were ligated into the pcDNA hygro^+^ expression vector using Bam HI and Not I sites. For propagation of plasmids, *Escherichia coli* XL1-Blue cells (Stratagene) were used.

#### Cell lines and transfection

HEK293T (DSMZ- German Collection of Microorganisms and Cell Cultures GmbH No.: ACC 635) and HeLa (DSMZ No.: ACC 57) cells were purchased from the Deutsche Sammlung Mikrobiologischer Zellen. All cells were cultured in humidified incubators in an atmosphere of 95% air and 5% CO_2_ in Dulbecco’s modified Eagle’s medium (Gibco) supplemented with 10% fetal calf serum (FCS) and penicillin (100 U/ml)/streptomycin (100 μg/ml). Transfection was carried out using polyethylenimine (PEI MAX Transfection Grade Linear Polyethylene, Polysciences) in a 2.5:1 PEI/DNA ratio.

### Western blotting

Western blotting was performed as described in (*54*). For lysis of cultured cells, cells were washed once with ice-cold phosphate-buffered saline (PBS) and then detached from the culture plate in PBS supplemented with cOmplete protease inhibitor mix (Roche) and 4 mM EDTA using a cell scraper. After centrifugation of the suspension at 3000 rpm and 4°C, the resulting cell pellet was resuspended in lysis buffer [150 mM NaCl, 1% (w/v) Triton X-100, 0.1% (w/v) SDS, and 50 mM tris-HCl (pH 7.4)] containing cOmplete protease inhibitor mix, pepstatin A (0.5 μg/ml; Sigma-Aldrich), 4 mM EDTA, and 4 mM Pefabloc SC Protease Inhibitor (Roth). After sonication, cells were allowed to lyse for 1 hour on ice before centrifugation at 13,000 rpm for 10 min. Protein containing supernatants were transferred into a fresh tube, and protein concentration was determined using the Pierce BCA Protein Assay Kit (Thermo Fisher Scientific) according to the manufacturer’s recommendations. For lysis of murine testes, testicles were decapsulated and blended in lysis buffer without any detergents. After blending, Triton X-100 and SDS were added in the concentration mentioned above, and lysis was continued as described before. After determination of their protein concentration, lysates were supplemented with the required amount of 5× SDS sample buffer [500 mM dithiothreitol (DTT), 5% (w/v) SDS, 50% (v/v) glycerol, trace amounts bromophenol blue, and 625 mM tris-HCl (pH 6.8)]. Equal protein amounts were separated using a classical tris-glycine buffer system using 7.5 to 12% polyacrylamide gels using the PageRuler Prestained Protein Ladder (Thermo Fisher Scientific) as reference marker. Following semidry transfer to nitrocellulose membranes, unspecific protein binding to the membranes was blocked using TBS-T [137 mM NaCl, 2.7 mM KCl, 0.1% (v/v) Tween 20, and 25 mM tris-HCl (pH 7.4)] supplemented with either 5% nonfat milk powder (Roth) or, in case of detection of detection of PLN, 5% BSA for at least 1 hour. Immunodetection was carried out as described in (*55*) using the following antibodies: anti-HA 3F10 (Roche), anti-myc 9B11 (Cell Signaling Technology), anti-myc 71D10 (Cell Signaling Technology), anti-FLAG (M2, Sigma-Aldrich), anti-PLN (2D12, Thermo Fisher Scientific), anti-Stx8 (AF5448, R&D), anti-Izumo1 (#125, BioAcademia), anti-ACE3 (ABIN2451916, antibodies-online.com), anti-SPPL2c (*20*), anti–lysosomal integral membrane protein (LIMP-2) (*56*), anti-SOF1 (IGX-46660, Imugex), anti-SOF1 (*13*), anti-cofilin (D3F9, Cell Signaling Technology), anti-actin (A2066, Sigma-Aldrich), anti–α-tubulin (2411S, Cell Signaling Technology), anti–glyceraldehyde-3-phosphate dehydrogenase (W17079, BioLegend), and anti-Elongation factor 2 (EEF2) (ab33523, Abcam). For generation of a C11orf94-specific antibody, rabbits were immunized with a peptide corresponding to the C terminus of murine C11orf94 (CRPLLSQAQQRKRDGPNMAD), and antibodies were purified from sera of the respective animals against the immobilized immunogen. Functionality of the antibody for IHC and Western blot applications was validated using knockout tissue as control as depicted in the respective figures. Similarly, a custom-made antibody targeting murine SPACA6 (epitope CLKDMKINYDERSYL) was ordered from GenScript. Testing of the antibody in Western blot application is depicted in fig. S6B.

### Endoglycosidase H treatment of cell lysates

Cells were lysed as described above for Western blot preparation. After measurement of protein concentration, 50 μg of protein was supplemented with 2.5 μl of denaturing buffer [6% (w/v) SDS and 400 mM DTT, in 50 mM NaPO_4_ (pH 7.5)] and the total volume was adjusted to 30 μl. After denaturation for 5 min at 56°C, samples were cooled on ice, and 20 μl of 5× endoglycosidase H buffer (250 mM sodium acetate, pH 5.5) was added, as well as 4 μl of cOmplete protease inhibitor mix. The total volume was adjusted to 100 μl using double-distilled water (ddH_2_O). The sample was split into two 40 μl aliquots, which were supplemented with 1 μl of endoglycosidase H (Roth) or ddH_2_O as control. Samples were incubated for 4 hours at 37°C under gentle shaking. The reaction was stopped by addition of 10 μl of 5× SDS sample buffer and subsequent denaturation at 56°C for 5 min. Samples were lastly analyzed by Western blotting as described above.

### Blue native PAGE

For analysis of Izumo1-containing complexes in testis lysates, decapsulated testes were homogenized in the Novex NativePAGE sample buffer (Thermo Fisher Scientific) supplemented with protease inhibitors and 0.5% digitonin (Thermo Fisher Scientific) for 30 min on ice. After centrifugation at 13,000 rpm for 10 min at 4°C, protein concentration was measured using the Pierce BCA Protein Assay (Thermo Fisher Scientific). A total of 100 μl of the lysates was supplemented with 5 μl of NativePAGE 5% G-250 Sample Additive (Thermo Fisher Scientific), and 15 μg of protein was loaded on a NativePAGE 3 to 12% bis-tris gel (Thermo Fisher Scientific). For control of the approximate molecular weight of separated protein complexes, 5 μl of the Native Mark Unstained Protein Standard (Thermo Fisher Scientific) was applied additionally. Gels were run for approximately 20 min at 150 V using the dark blue cathode buffer, which was subsequently replaced with the light blue cathode buffer for the remaining separation. After electrophoresis, protein complexes were transferred to polyvinylidene difluoride membranes (Thermo Fisher Scientific) as described above but using 1× concentrated NuPAGE transfer buffer (Thermo Fisher Scientific). After blotting, proteins were fixed on the membrane using 8% acetic acid for 15 min and air-dried subsequently. The dried membranes were lastly detained by repeated washing steps with methanol and ddH_2_O and processed further for Western blot development as explained above.

### Coupling of C11orf94-directed antibody to Sepharose

The custom-made C11orf94 antibody described above was coupled to CNBr-activated Sepharose 4B (GE Healthcare) according to the manufacturer’s recommendations. Briefly, the C11orf94-targeting antibody–containing solution was desalted using PD-10 desalting columns (GE Healthcare) to remove tris ions from the respective solutions. The sample was eluted with 3.5 ml of coupling buffer [0.1 M NaHCO_3_ and 0.5 M NaCl (pH 8.3)], generating 0.5-ml fractions, which were subsequently monitored for their protein concentration using a NanoDrop 2000c Spectrophotometer (Thermo Fisher Scientific). Subsequently, the protein-containing fractions were pooled for further handling. A total of 2 mg of antibody was coupled to 1 ml of CNBr-activated Sepharose 4B in coupling buffer for 1.5 hours at room temperature (RT) under constant rotation. Subsequently, the beads were washed with coupling buffer and incubated with quenching solution (0.1 M tris-HCl, pH 8.0) for 2 hours at RT. Afterward, beads were washed with 0.1 M acetic acid, 0.5 M NaCl (pH 4.0), and 0.1 M tris-HCl, and 0.5 M NaCl (pH 8.0) in three repetitive cycles. Last, beads were resuspended in the same amount of 50 mM tris (pH 7.4) supplemented with 0.01% sodium azide. Control beads for preclearing of the lysates were generated by incubation of HCl-activated Sepharose with 0.1 M tris-HCl (pH 8) for 2 hours instead of the antibody solution.

### Coimmunoprecipitation

For coimmunoprecipitation, cells or tissues were lysed in lysis buffer containing either 1% (w/v) Triton X-100 (Sigma-Aldrich) or 0.5% CHAPSO (Roth) as described above. After lysis, equal protein amounts were supplemented with the antibodies indicated in the respective figures and incubated at 4°C overnight under continuous rotation. Subsequently, 25 μl of protein G agarose was added to each lysate and incubated at 4°C for 2 hours with constant rotation. The beads were sedimented by centrifugation at 1500g for 2 min and washed five times with the respective IP buffer. After the final washing step, precipitated proteins were eluted from the beads using 60 μl of SDS sample buffer for 10 min at 56°C. Samples were spun for 5 min at 13,000 rpm and analyzed by gel electrophoresis. Alternatively, lysates were incubated with 25 μl anti-FLAG M2 agarose gel (Sigma-Aldrich) or anti-C11orf94 Sepharose beads overnight at 4°C and then processed for washing as described above. In case of precipitation of Izumo1 for the analysis of its interaction with ACE3, the Izumo1-directing antibody was rebuffered to coupling buffer using Amicon Ultra 0.5-ml centrifugal filter units (Sigma-Aldrich) with a 30-kDa molecular weight cut off. The purified antibodies were then coupled to agarose beads using a Pierce Direct IP kit (Thermo Fisher Scientific).

### Mass spectrometric identification of C11orf94 interaction partners

C11orf94-interacting proteins were precipitated as describe above using anti-C11orf94 Sepharose beads. After two subsequent washes with IP buffer without detergent, beads were resuspended in 100 μl of 20 mM tris-HCl (pH 7.4). Samples were digested on beads twice with 200 ng of trypsin overnight (Trypsin Gold, Promega, USA/Germany) and subsequently with 100 ng of rLys-C (Promega, USA/Germany) overnight. Protein digests were desalted with Ultra-Micro C18 SpinColumns (Harvard Apparatus, USA). Last, dried digests were recovered with 3 μl of 30% formic acid in water supplemented with standard peptide mixture (25 fmol/μl; Retention Time Calibration Mixture, Thermo Pierce, Germany) and diluted to a final volume of 23 μl with water; a total of 5 μl of this solution was injected for nano liquid chromatography–tandem MS (nanoLC-MS/MS). NanoLC-MS/MS analyses were performed with a Q Exactive HF mass spectrometer (Thermo Fisher Scientific, USA/Germany) hyphenated to Nanoflow LC system (Dionex 3000 RSLC, Thermo Dionex, Germany). Peptides were separated in linear gradient of 0.1% aqueous formic acid (eluent A) and 0.1% formic acid in 60% acetonitrile (eluent B) in 120 min, and the mass spectrometer was operated in data-dependent acquisition mode (TopN 10). Raw files were imported into Progenesis QIP v4.2 (Nonlinear Dynamics, UK) software for analysis and quantification. Peptide and protein identifications were performed with Mascot v2.6 (Matrix Science, UK). Fold changes were calculated in Microsoft Excel. Detailed data about instrument settings are provided in Supplementary Text.

### Indirect immunofluorescence

Transiently transfected HeLa cells grown on coverslips were washed twice with PBS and fixed with 4% p-formaldehyde in PBS for 20 min at RT. Afterward, cells were washed three times with PBS/0.2% saponin (Roth) three times and incubated for 5 min with 0.12% glycine/PBS supplemented with 0.2% saponin. Samples were blocked for 1 hour in blocking buffer (1% FCS in PBS/0.2% saponin) and subsequently incubated with primary antibodies as indicated in the respective figures at 4°C overnight. Antibodies against the organelle marker proteins calnexin (2679T, Cell Signaling Technology), GM130 (clone 35/GM130, BD Biosciences), and ERGIC53 (OTI1A8, Enzo Life Sciences) were used to evaluate the subcellular localization of C11orf94. The other used antibodies have been described in the Western blotting section. After five washes, coverslips were incubated with secondary antibodies coupled to either Alexa Fluor 488 or Alexa Fluor 594 (Molecular Probes) diluted in blocking buffer for 1 hour at RT. Last, coverslips were washed five times with PBS/saponin and twice with ddH_2_O and mounted on slides using a mixture of Mowiol/1,4-diazabicyclo[2.2.2]octane (DABCO) containing 4′,6-diamidino-2-phenylindole (DAPI) (1 μg/ml) for visualization of nuclei. Samples were analyzed using either an Olympus FV1000 or Leica SP5 confocal laser scanning microscope. Pictures were processed using ImageJ and GNU Image Manipulation Program software.

### Staining of sperm cells

Sperm cells were isolated as described above, and 4 μl of the resulting sperm suspension was fixed in a drop of 4% paraformaldehyde. After drying on a histology-suitable slide, cells were further processed as described above for indirect immunofluorescence. After incubation with the secondary antibody, slides were washed three times with PBS/saponin and stained with PNA-FITC (20 μg/ml; Sigma-Aldrich) for 30 min in the dark. Subsequently, slides were washed, and cells were mounted in Mowiol/DABCO/DAPI as described above.

### Staining of testis sections

After dissection, whole testes were fixed in Bouin’s fixative (Sigma-Aldrich) for 24 hours at 4°C and then incubated in 30% sucrose in 0.1 M phosphate buffer [16 mM KH_2_PO_4_ and 84 mM Na_2_HPO_4_ (pH 7.4)] overnight at 4°C. Subsequently, testes were washed twice with phosphate buffer and embedded with Epredia Cryomatrix embedding resin (Thermo Fisher Scientific) and frozen on dry ice. Two-micrometer thin sections were prepared using a Leica CM1900 cryostat and dried for 1 hour on HistoBond+M adhesive microscope slides (VWR). After two washes in phosphate buffer, slides were boiled for 30 min in citrate buffer (10 mM Na-citrate, pH 6.0) and allowed to cool to RT. Afterward, slides were washed three times with phosphate buffer containing 0.25% Triton X-100 and incubated for at least 1 hour in blocking solution (phosphate buffer, 0.25% Triton X-100, and 5% FCS). Subsequently, the antibodies required for staining were diluted in blocking solution and incubated together with the samples overnight. For analysis of the ER localization of Izumo1 in the murine testis, the organelle was labeled with anti-BIP (C50B12, Cell Signaling Technology). After three washes for 10 min with phosphate buffer + 0.25% Triton X-100, the respective secondary antibodies conjugated with either Alexa Fluor 488 or Alexa Fluor 594 (both from Thermo Fisher Scientific) dyes were administered for 1 hour at RT. After three washes with phosphate buffer + 0.25% Triton X-100, samples were rinsed with ddH_2_O twice and embedded with Mowiol/DABCO/DAPI. Samples were analyzed using a Leica SP5 confocal laser scanning microscope. Pictures were processed using ImageJ and GNU Image Manipulation Program software.

### RNA isolation and quantitative PCR

RNA was isolated from murine testis using the NucleoSpin RNA Plus Kit (Macherey-Nagel). A total of 1000 ng of RNA was subsequently transcribed into cDNA using the RevertAid First Strand cDNA Synthesis Kit (Thermo Fisher Scientific). Quantitative PCR–based analysis of transcript levels was performed according to (*57*) using the Universal ProbeLibrary System (Roche) and a LightCycler 480II (Roche) or CFX384 Touch Real-Time PCR Detection System (Bio-Rad) using the following primers: mC11orf94 forward (probe 3), 5′-TCAGGCCTGGTGGATGAC-3′ and mC11orf94 reverse (probe 3), 5′-TCATAGTAATAGTCAGCCATGTTGG-3′ and mTuba1a forward (probe 88), 5′-CTGGAACCCACGGTCATC-3′ and mTuba1a reverse (probe 88), 5′-TGTAGTGGCCACGAGCATAG-3′.

### Surface biotinylation

Transiently transfected HeLa cells were washed twice with PBS-CM [PBS + 0.1 mM CaCl_2_ + 1 mM MgCl_2_ (pH 8.0)] and treated with EZ-Link Sulfo-NHS-SS-Biotin (1 mg/ml; Thermo Fisher Scientific) in PBS-CM for 30 min. Subsequently, cells were washed once with PBS-CM, and the remaining biotin was quenched using 50 mM tris/PBS (pH 8.0) for 10 min at 4°C. After two washes with PBS, cell lysates were prepared as described above. Biotinylated proteins were recovered using High Capacity Streptavidin Agarose beads (Thermo Fisher Scientific) for 2 hours at 4°C under continuous rotation. Last, proteins were eluted from the beads using 60 μl of SDS sample buffer and analyzed by Western blotting.

### Cellular fractionation

Transfected HEK293T cells were washed twice with PBS, followed by two additional washes with homogenization buffer [250 mM sucrose, 1 mM EDTA, and 10 mM Hepes-NaOH (pH 7.4)]. Afterward, cells were scraped off in 1 ml of homogenation buffer supplemented with protease inhibitors. A total of 50 μl of the cell suspension was transferred into a fresh tube for preparation of a total lysate as described above. The remaining cells were spun down at 1000*g* for 5 min at 4°C and resuspended in 500 μl of hypotonic buffer [1 mM EDTA and 10 mM Hepes-NaOH (pH 7.4)] supplemented with protease inhibitors. After 5 min on ice, cells were passed five times through a 27-gauge cannula. Subsequently, 500 μl of a 2× sucrose buffer [500 mM sucrose, 1 mM EDTA, and 10 mM Hepes-NaOH (pH 7.4)] was added, and the procedure was repeated. Afterward, postnuclear supernatants were prepared by centrifugation at 750*g* for 5 min. The resulting supernatants were subjected to ultracentrifugation at 100,000*g* at 4°C for 1 hour using an Optima Max Ultracentrifuge (Beckman Coulter). A total of 200 μl of the resulting supernatant was saved as cytosolic fraction; the pellet was resuspended in 50 μl of resuspension buffer (10 mM Hepes-NaOH, pH 7.4). Afterward, 650 μl of ddH_2_O and 700 μl of 2× carbonate solution (200 mM sodium carbonate, pH 11.5) were added to remove non-integral membrane proteins. After 1 hour, the membrane suspension was centrifuged again for 1 hour at 100,000*g*. After one additional washing step with 1 ml of resuspension buffer, the remaining membrane pellet was lastly dissolved in 100 μl of lysis buffer supplemented with protease inhibitors. The protein concentration of total lysates and cytosolic and membrane fractions was lastly measured as described above.

### Statistical analysis

Calculations were carried out using Excel 2016. GraphPad Prism Software was used to generate diagrams and to calculate statistical significance. Most data were normalized to the mean of the respective control sample (EV transfected or wild type) to calculate fold changes. All diagrams depict mean values ± SD; statistical tests underlying the indicated significance values are indicated in the individual figure legends. *N* indicates the number of independent experiments, while *n* refers to the total number of samples/animals tested for the respective dataset. All coimmunoprecipitation and immunofluorescence experiments that were not quantified and where the number of repetitions is not indicated in the respective figure legends were at least performed twice.

## Supplementary Materials

### This PDF file includes:

Supplementary Text Figs. S1 to S6 References

## Notes

View/request a protocol for this paper from *Bio-protocol*.
