# Supplementary material for: C11orf94/Frey is a key regulator for male fertility by controlling Izumo1 complex assembly
Source: Sci Adv. 2022 Aug 12;8(31):eabo6049. doi: 10.1126/sciadv.abo6049 (PMC9374335; doi:10.1126/sciadv.abo6049)
Supplement: Supplementary file 1 — Supplementary Text Figs. S1 to S6 References [file sciadv.abo6049_sm.pdf]

Science Advances
AAAS

Supplementary Materials for

C11orf94/Frey is a key regulator for male fertility by controlling Izumo1 complex assembly

Whendy Contreras *et al.*

Corresponding author: Torben Mentrup, torben.mentrup@tu-dresden.de

Sci. Adv. **8**, eabo6049 (2022)
DOI: 10.1126/sciadv.abo6049

This PDF file includes:

Supplementary Text
Figs. S1 to S6
References

Supplementary Text

Instrumentation, parameters and software for mass spectrometry

Q-EXACTIVE HF

Instrument / Parameter	Value	Comments
**Q-Exactive HF**	ThermoScientific, Bremen, Germany	DDA-Mode (positive ion mode)
**MS1**		
Polarity	positive	
Resolution	R120000 at m/z 200	
AGC	3x 10E6	
Max. Fill Time	100ms	
Lock Mass	m/z 445.120025	Dodecamethylcyclohexasiloxane *(60)*
Scan Range	m/z 395-1500	
Picotip Needle	20µm / 10µm	NewObjectives, Ithaca, USA
Voltage	2.3-2.7kV	(might vary between experiments)
**MS2 High Res**	Top10	**HCD**
Resolution	R15000 at m/z 200	
AGC	1E5	
Max. Fill Time	50ms	
Isolation	2.0 m/z	
Isolation window Offset	0.3 m/z	
Scan Range	200-2000 m/z	
Fixed 1st Mass	-	
Norm. Collision Energy	27	
Threshold	2E3 / 4E4	
Charge states	Unassigned, 1, 6-8, >8	(rejected)
Dynamic Exclusion	15s / 3ppm	

Thermo Dionex3000 RSLC

Instrument / Material	Manufacturer (Supplier)	Comments
**Dionex3000 RSLC**	ThermoScientific, Idstein, Germany	Nanoflow System
Acclaim PepMap 100 C18, 3 µm, 300 µm x 5 mm, Acclaim PepMap C18 3, µm, 75 µm x 15 cm	ThermoScientific, Idstein, Germany	Trap-Column Setup<br>Load: 2µl/min<br>Separation: 200nl/min

Software

Instrument / Material	Manufacturer (Supplier)	Comments
MASCOT V2.6 *(61)*	MatrixScience, London, UK	Protein Identification Software matrixscience.com
Progenesis QIP V4.2	Nonlinear Dynamics, Newcastle u.T., UK	Quantitative data interpretation (Peak Picking, MS/MS Export, Peptide ID import and Assignment, Quantification (MI3/Hi3))

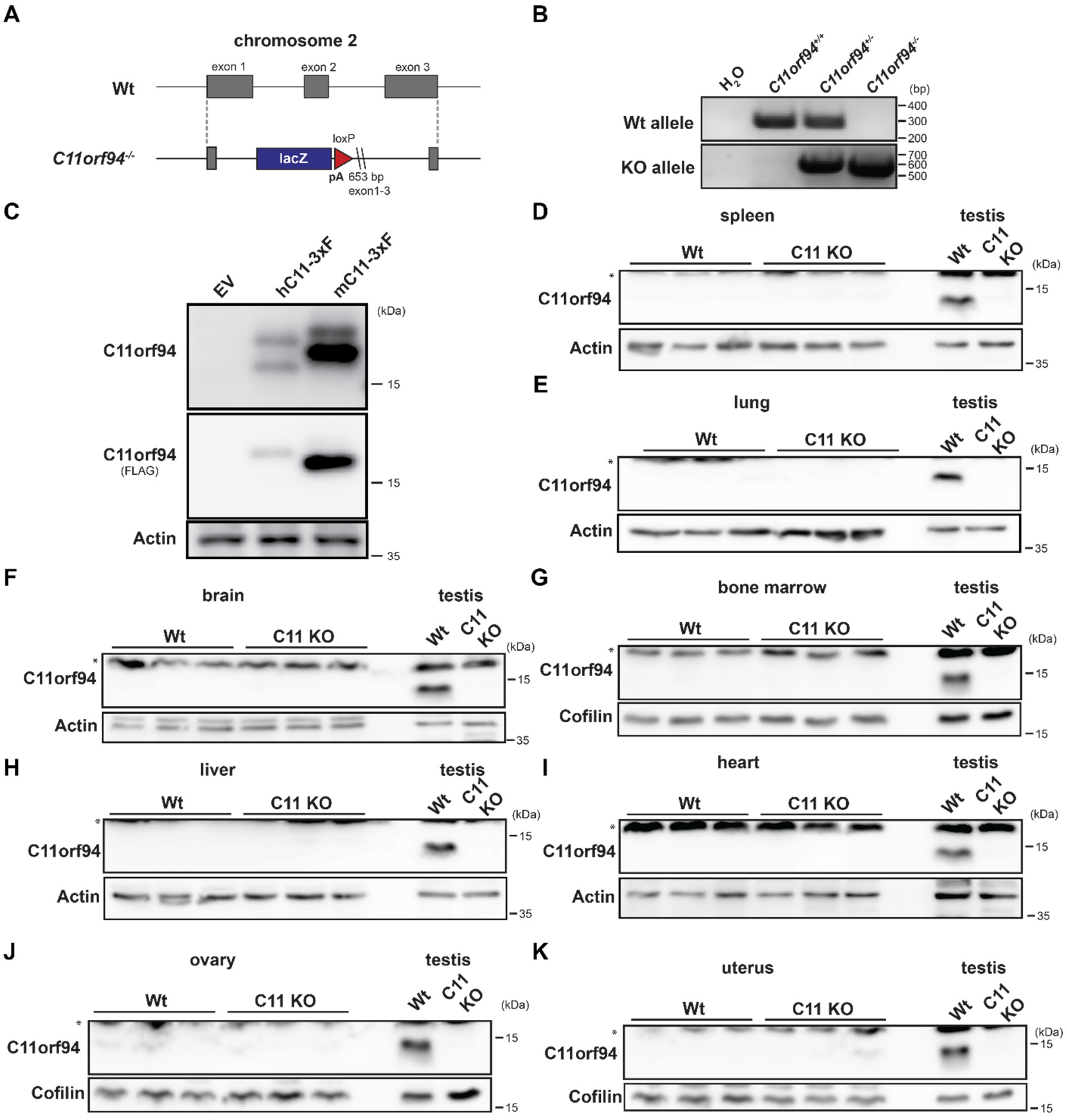

Fig. S1. C11orf94 is exclusively expressed in the murine testis. (A) Targeting strategy for generation of the *C11orf94*$^{-/-}$ strain. The complete protein-coding region of the wild type (Wt) *C11orf94* allele was deleted and a LacZ reporter cassette was integrated in this locus. **(B)** Representative example for genotyping PCRs detecting either the wild type or knockout allele. **(C)** HEK cells were transfected with either human (h) or murine (m) C11orf94-3xFLAG. Lysates of these cells were subsequently employed to test the newly generated C11orf94 antibody targeting the C-terminus of the murine protein. Anti-FLAG served as control. Expression of C11orf94 was analysed in spleen **(D)**, lung **(E)**, brain **(F)**, bone marrow **(G)**, liver **(H)**, heart **(I)**, ovary **(J)** and uterus **(K)** of wild type mice by Western Blotting. While lysates from the respective organs isolated from C11orf94-deficient mice (C11 KO) served as negative control, a wild type testis lysate was

included in the analysis as positive control. The asterisk indicates an unspecific protein band stained with the C11orf94 antibody.

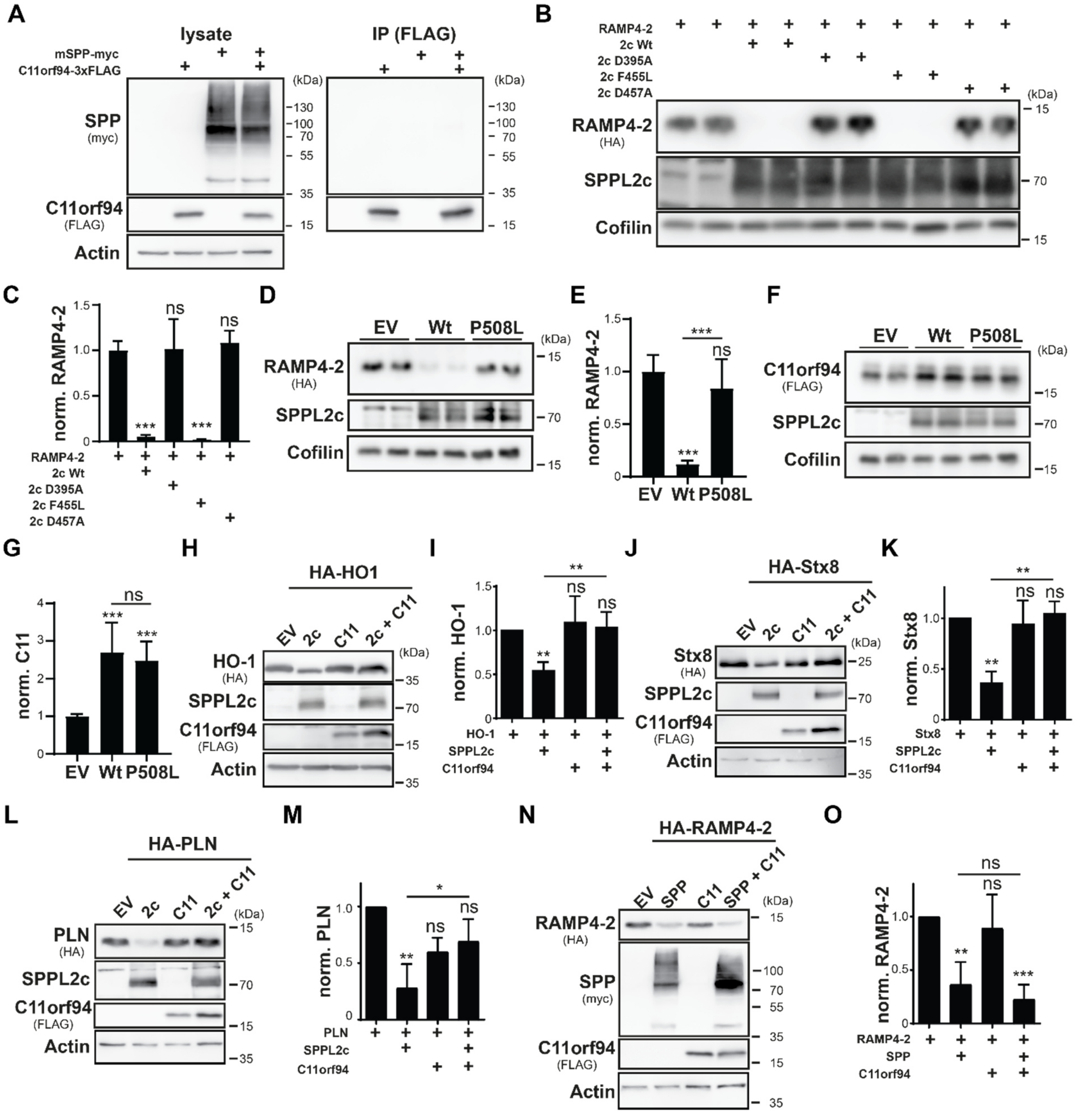

Fig. S2. C11orf94 specifically inhibits SPPL2c-mediated proteolysis. (A) HEK cells were transiently transfected with mC11orf94-3xFLAG and mSPP-myc and interaction of both proteins was analysed by co-immunoprecipitation. For this purpose, C11orf94 was precipitated from lysates (1% Triton X100) of these cells using anti-FLAG and protein G agarose. Bead eluates (IP) as well as total lysates were finally subjected to Western Blotting. **(B)** To test proteolytic activity of SPPL2c active site mutants, HEK cells were transfected with HA-RAMP4-2 and the indicated SPPL2c mutants or an empty vector (EV) as control. RAMP4-2 proteolysis was finally evaluated by immunoblotting. **(C)** Quantification of B). N=2, n=4, One-Way ANOVA with Tukey's *post hoc* test. Statistical indices above the bars indicate significance against the EV-transfected control. **(D)** Proteolysis of RAMP4-2 by SPPL2c P508L in transfected HEK cells was analysed by Western Blotting. **(E)** Quantification of D). N=3, n=6, One-Way ANOVA with Tukey's *post hoc* test.

Throughout the Figure, statistical indices above the bars depict significance against the EV control, further comparisons are indicated with lines. **(F)** HEK cells transiently transfected with the indicated constructs were lysed and C11orf94 protein levels were compared by Western Blotting. **(G)** Quantification of F). N=4, n=8, One-Way ANOVA with Tukey's *post hoc* test. **(H)** HEK cells were transfected with HA-HO1 alone or in different combinations with mSPPL2c-myc (2c) and/or mC11orf94-3xFLAG (C11). Processing of HA-HO1 by SPPL2c under these conditions was visualised by Western Blotting following lysis of the cells. **(I)** Quantification of H). N=5, n=5, One-Way ANOVA with Tukey's *post hoc* test. **(J)** Same as described in H) but employing HA-Stx8 as substrate. **(K)** Quantification of J). N=3, n=3, One-Way ANOVA with Tukey's *post hoc* test. **(L)** Proteolysis of HA-mPLN was analysed as described in H) for HA-HO1. (M) Quantification of L). N=3, n=3, One-Way ANOVA with Tukey's *post hoc* test. **(N)** Regulation of SPP-mediated intramembrane proteolysis of HA-RAMP4-2 by C11orf94 was evaluated by Western Blot analysis of HEK cells transiently transfected with the indicated constructs. **(O)** Quantification of N). N=4, n=4, One-Way ANOVA with Tukey's *post hoc* test. ns, not significant; * $p \leq 0.05$; ** $p \leq 0.01$; *** $p \leq 0.001$.

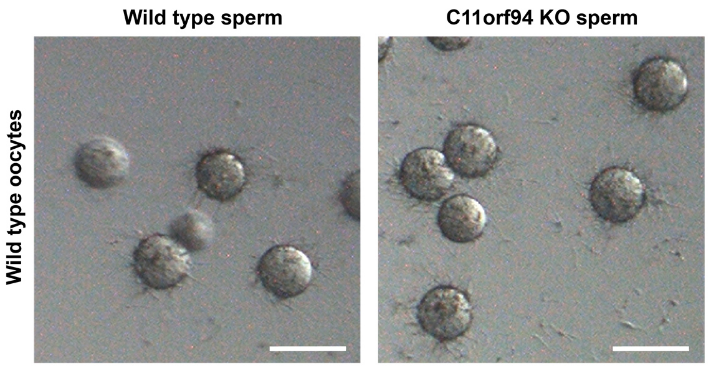

Fig. S3. Loss of C11orf94 does not impair binding of sperm to oocytes. Binding of sperm cells from either wild type or C11orf94-deficient mice (C11orf94 KO) to wild type oocytes without zona pellucida was monitored 1 h after IVF by light microscopy. Scale bar, 100 µm.

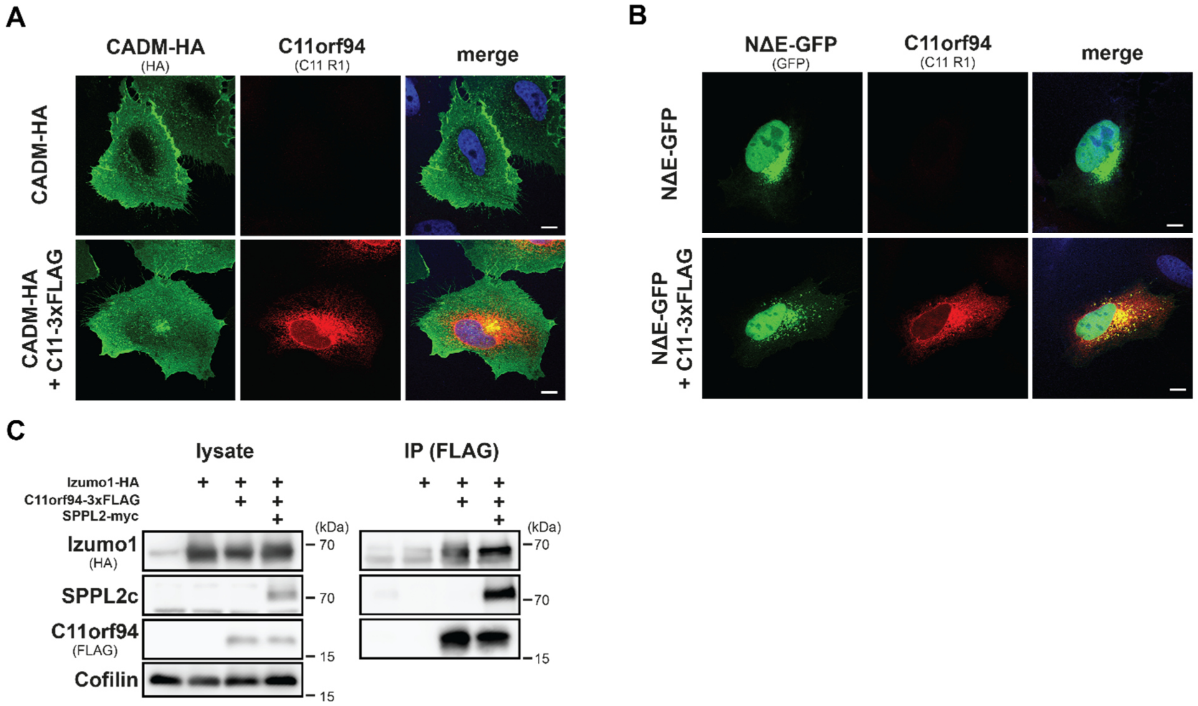

Fig. S4. C11orf94 specifically regulates Izumo1 trafficking. (A) HeLa cells were transiently transfected with mCADM-HA alone or in combination with mC11orf94-3xFLAG. After PFA-fixation, localisation of both proteins was subsequently visualised by indirect immunofluorescence employing the indicated antibodies. Scale bar, 10 µm. **(B)** Same as in A) but employing Notch1ΔE-eGFP (NΔE-GFP) instead of CADM-HA. Scale bar, 10 µm. **(C)** Interaction of Izumo1-HA and C11orf94-3xFLAG was analysed in presence or absence of SPPL2c-myc. HeLa cells transiently transfected with the indicated constructs were lysed in 0.5% CHAPSO and C11orf94-containing complexes were precipitated using anti-FLAG beads. Presence of the individual proteins in total lysates and bead eluates (IP) was finally evaluated by Western Blotting.

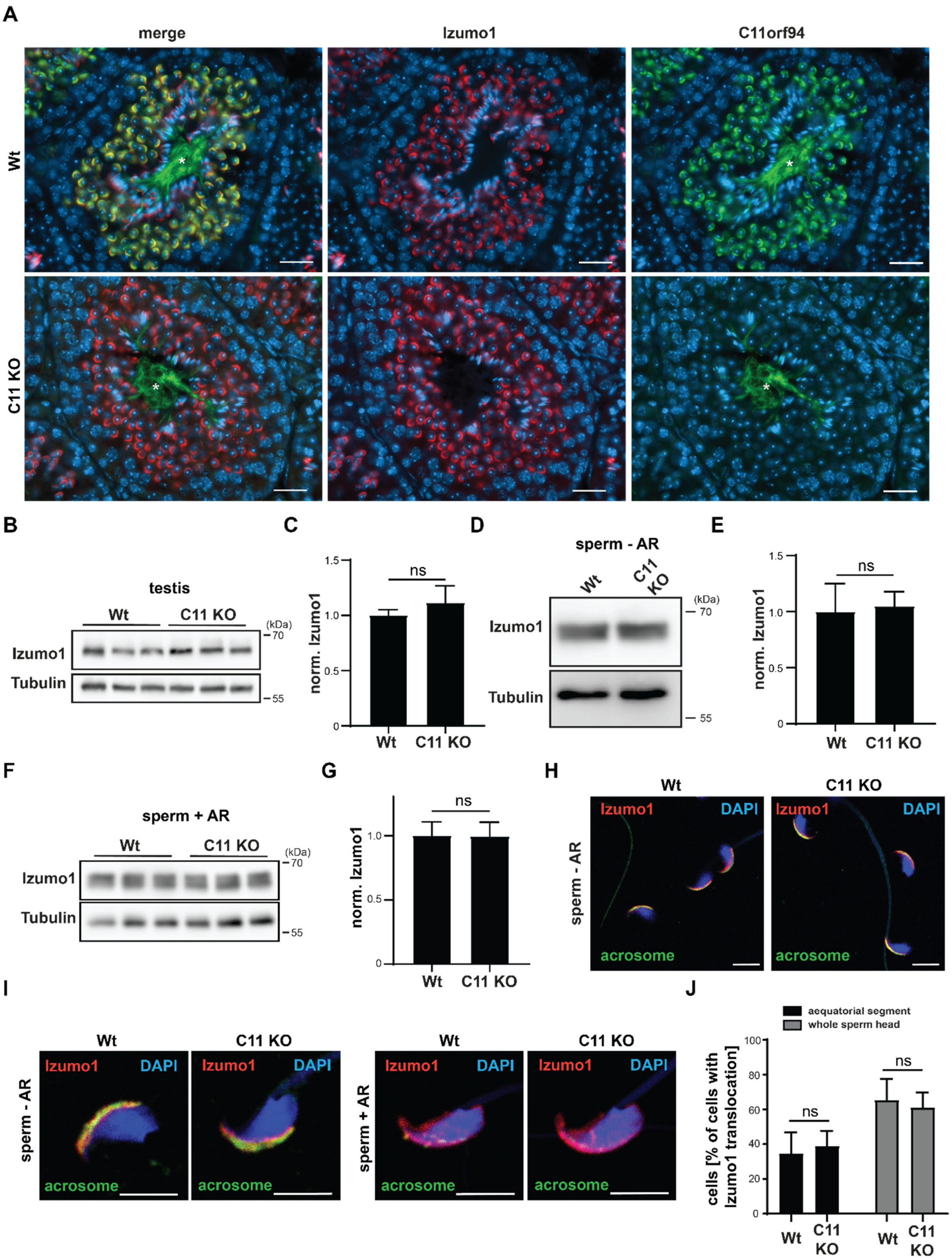
A
merge
Izumo1
C11orf94
Wt
C11 KO
B
testis
Wt
C11 KO
Izumo1
Tubulin
(kDa)
70
55
C
norm. Izumo1
ns
Wt
C11 KO
D
sperm - AR
Wt
C11 KO
Izumo1
Tubulin
(kDa)
70
55
E
norm. Izumo1
ns
Wt
C11 KO
F
sperm + AR
Wt
C11 KO
Izumo1
Tubulin
(kDa)
70
55
G
norm. Izumo1
ns
Wt
C11 KO
H
Wt
C11 KO
sperm - AR
Izumo1
DAPI
acrosome
I
Wt
C11 KO
sperm - AR
sperm + AR
Izumo1
DAPI
acrosome
J
aequatorial segment
whole sperm head
cells [% of cells with Izumo1 translocation]
ns
Wt
C11 KO

Fig. S5. Loss of C11orf94 does not impact on acrosomal sorting of Izumo1. (A) Localisation of Izumo1 and C11orf94 was analysed in testis cryosections from either wild type (Wt) or C11-deficient mice (C11 KO) mice by immunohistochemistry. Nuclei were stained with DAPI. Scale bar, 25 µm. **(B)** Izumo1 protein levels in the testis were compared between Wt and C11 KO mice by Western Blotting. **(C)** Quantification of B). N=2, n=6, two-tailed unpaired Student's t-test. **(D)** Non-capacitated epididymal spermatozoa not subjected to acrosome reaction (-AR) from Wt or C11 KO mice were analysed for Izumo1 protein levels by immunoblotting. **(E)** Quantification of D). N=3, n=7(Wt)/6(C11 KO), two-tailed unpaired Student's t-test. **(F)** The experiment described in D) was repeated using sperm cells that were first capacitated for 45 min and then subjected to treatment with 10 µM Calcimycin to induce the acrosome reaction (+AR). **(G)** Quantification of F). N=1, n=3, two-tailed unpaired Student's t-test. Localisation of Izumo1 in non-capacitated **(H)** or capacitated and acrosome-reacted **(I)** epididymal spermatozoa from Wt or C11orf94 KO mice was analysed by indirect immunofluorescence. Acrosome reaction was induced by treatment with 10 µM Calcimycin for 45 min. Sperm cells were fixed with PFA prior to staining of Izumo1 using a specific antibody as well as acrosomes employing PNA-FITC and nuclei with DAPI. Scale bar, 5 µm. **(J)** The localisation of Izumo1 in sperm cells from Wt or C11 KO mice that showed a clear redistribution of Izumo1 upon induction of the acrosome reaction was categorised as either within the aequatorial segment or spread over the whole sperm head. At least 49 sperm cells per mouse were analysed. N=2, n=5(Wt)/6(C11 KO), two-tailed unpaired Student's t-test. ns, not significant.

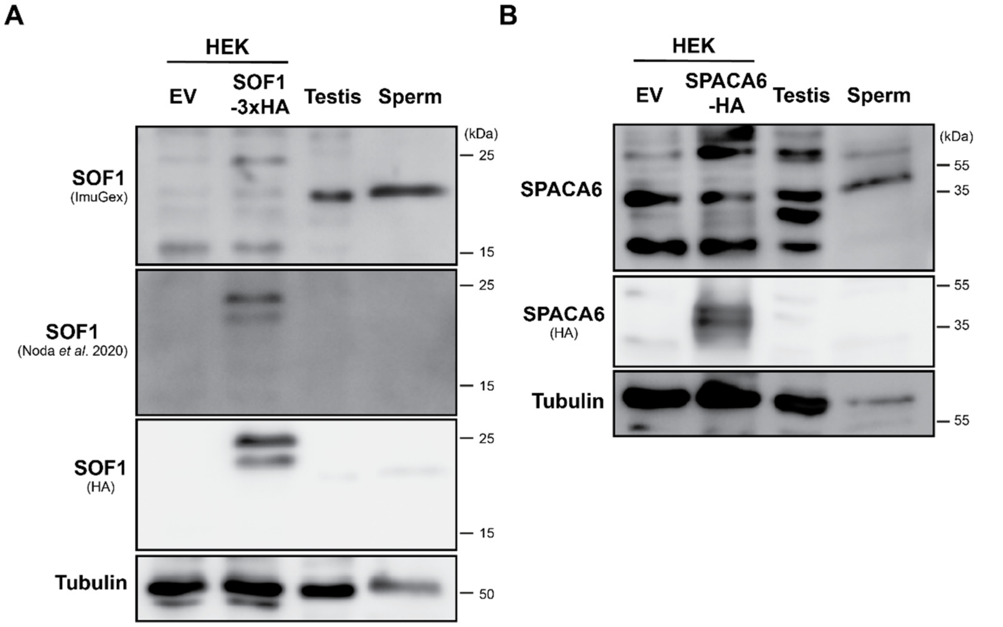

Fig. S6. Validation of antibodies targeting SOF1 or SPACA6. (A) HEK cells transfected with an empty vector (EV) or SOF1-3xHA as well as testis and sperm cells from a wild type mouse were lysed. Antibodies targeting SOF1 were subsequently tested by Western Blotting for detection of the endogenous or overexpressed protein. Expression of SOF1-3xHA was validated employing an antibody targeting the HA epitope. Tubulin served as loading control. **(B)** A custom-made antibody targeting murine SPACA6 was validated as described in A) but employing HEK cells transfected with SPACA6-HA as control.

REFERENCES AND NOTES

Commented [FB1]: References 46 and 47 were identical; thus, Ref. 47 was deleted and citations were renumbered accordingly. Please check renumbering.

1. E. Bianchi, G. J. Wright, Find and fuse: Unsolved mysteries in sperm-egg recognition. *PLOS Biol.* **18**, e3000953 (2020).

2. H. H. Bhakta, F. H. Refai, M. A. Avella, The molecular mechanisms mediating mammalian fertilization. *Development* **146**, dev176966 (2019).

3. A. R. Krauchunas, M. R. Marcello, A. Singson, The molecular complexity of fertilization: Introducing the concept of a fertilization synapse. *Mol. Reprod. Dev.* **83**, 376–386 (2016).

4. N. Inoue, M. Ikawa, A. Isotani, M. Okabe, The immunoglobulin superfamily protein Izumo is required for sperm to fuse with eggs. *Nature* **434**, 234–238 (2005).

5. N. Sebkova, L. Ded, K. Vesela, K. Dvorakova-Hortova, Progress of sperm IZUMO1 relocation during spontaneous acrosome reaction. *Reproduction* **147**, 231–240 (2014).

6. M. Chalbi, V. Barraud-Lange, B. Ravaux, K. Howan, N. Rodriguez, P. Soule, A. Ndzoudi, C. Boucheix, E. Rubinstein, J. P. Wolf, A. Ziyyat, E. Perez, F. Pincet, C. Gourier, Binding of sperm protein Izumo1 and its egg receptor Juno drives Cd9 accumulation in the intercellular contact area prior to fusion during mammalian fertilization. *Development* **141**, 3732–3739 (2014).

7. T. Saito, I. Wada, N. Inoue, Sperm IZUMO1-dependent gamete fusion influences male fertility in mice. *Int. J. Mol. Sci.* **20**, 4809 (2019).

8. E. Bianchi, B. Doe, D. Goulding, G. J. Wright, Juno is the egg Izumo receptor and is essential for mammalian fertilization. *Nature* **508**, 483–487 (2014).

9. C. Jean, F. Haghighirad, Y. Zhu, M. Chalbi, A. Ziyyat, E. Rubinstein, C. Gourier, P. Yip, J. P. Wolf, J. E. Lee, C. Boucheix, V. Barraud-Lange, JUNO, the receptor of sperm IZUMO1, is expressed by the human oocyte and is essential for human fertilisation. *Hum. Reprod.* **34**, 118–126 (2019).

10. U. Ohto, H. Ishida, E. Krayukhina, S. Uchiyama, N. Inoue, T. Shimizu, Structure of IZUMO1-JUNO reveals sperm-oocyte recognition during mammalian fertilization. *Nature* **534**, 566–569 (2016).

11. V. E. Deneke, A. Pauli, The fertilization enigma: How sperm and egg fuse. *Annu. Rev. Cell Dev. Biol.* **37**, 391–414 (2021).

12. N. G. Brukman, K. P. Nakajima, C. Valansi, X. Li, T. Higashiyama, B. Podbilewicz, IZUMO1 is a sperm fusogen. bioRxiv 478669 [**Preprint**]. 2022; https://doi.org/10.1101/2022.02.01.478669.

13. T. Noda, Y. Lu, Y. Fujihara, S. Oura, T. Koyano, S. Kobayashi, M. M. Matzuk, M. Ikawa, Sperm proteins SOF1, TMEM95, and SPACA6 are required for sperm-oocyte fusion in mice. *Proc. Natl. Acad. Sci. U.S.A.* **117**, 11493–11502 (2020).

14. D. A. Ellerman, J. Pei, S. Gupta, W. J. Snell, D. Myles, P. Primakoff, Izumo is part of a multiprotein family whose members form large complexes on mammalian sperm. *Mol. Reprod. Dev.* **76**, 1188–1199 (2009).

15. N. Inoue, Y. Hagihara, D. Wright, T. Suzuki, I. Wada, Oocyte-triggered dimerization of sperm IZUMO1 promotes sperm-egg fusion in mice. *Nat. Commun.* **6**, 8858 (2015).

16. A. S. Gaikwad, A. L. Anderson, D. J. Merriner, A. E. O'Connor, B. J. Houston, R. J. Aitken, M. K. O’Bryan, B. Nixon, GLIPR1L1 is an IZUMO-binding protein required for optimal fertilization in the mouse. *BMC Biol.* **17**, 86 (2019).

17. S. Barbaux, C. Ialy-Radio, M. Chalbi, E. Dybal, M. Homps-Legrand, M. Do Cruzeiro, D. Vaiman, J.-P. Wolf, A. Ziyyat, Sperm SPACA6 protein is required for mammalian sperm-egg adhesion/f-usion. *Sci. Rep.* **10**, 5335 (2020).

18. N. Inoue, Y. Hagihara, I. Wada, Evolutionarily conserved sperm factors, DCST1 and DCST2, are required for gamete fusion. *eLife* **10**, e66313 (2021).

19. I. Lamas-toranzo, J. G. Hamze, E. Bianchi, B. Fernández-fuertes, S. Pérez-Cerezales, R. Laguna-barraza, R. Fernández-González, P. Lonergan, A. Gutiérrez-Adán, G. J. Wright, M. Jiménez-Movilla, P. Bermejo-Álvarez, TMEM95 is a sperm membrane protein essential for mammalian fertilization. *eLife* **9**, e53913 (2020).

20. J. Niemeyer, T. Mentrup, R. Heidasch, S. A. Muller, U. Biswas, R. Meyer, A. A. Papadopoulou, V. Dederer, M. Haug-Kroper, V. Adamski, R. Lullmann-Rauch, M. Bergmann, A. Mayerhofer, P. Saftig, G. Wennemuth, R. Jessberger, R. Fluhrer, S. F. Lichtenthaler, M. K. Lemberg, B. Schroder, The intramembrane protease SPPL2c promotes male germ cell development by cleaving phospholamban. *EMBO Rep.* **20**, e46449 (2019).

21. T. Mentrup, B. Schröder, Signal peptide peptidase-like 2 proteases: Regulatory switches or proteasome of the membrane? *Biochim. Biophys. Acta Mol. Cell Res.* **1869**, 119163 (2022).

22. R. Kopan, M. X. Ilagan, γ-Secretase: Proteasome of the membrane? *Nat. Rev. Mol. Cell Biol.* **5**, 499–504 (2004).

23. G. Güner, S. F. Lichtenthaler, The substrate repertoire of γ-secretase/presenilin. *Semin. Cell Dev. Biol.* **105**, 27–42 (2020).

24. A. A. Papadopoulou, S. A. Müller, T. Mentrup, M. D. Shmueli, J. Niemeyer, M. Haug-Kröper, J. von Blume, A. Mayerhofer, R. Feederle, B. Schröder, S. F. Lichtenthaler, R. Fluhrer, Signal peptide peptidase-like 2c (SPPL2c) impairs vesicular transport and cleavage of SNARE proteins. *EMBO Rep.* **20**, e46451 (2019).

25. J.-A. Mäkelä, S. Cisneros-Montalvo, T. Lehtiniemi, O. Olotu, H. M. La, J. Toppari, R. M. Hobbs, M. Parvinen, N. Kotaja, Transillumination-assisted dissection of specific stages of the mouse seminiferous epithelial cycle for downstream immunostaining analyses. *J. Vis. Exp.* **164**, e61800 (2020).

26. E. Bianchi, G. J. Wright, Izumo meets Juno: Preventing polyspermy in fertilization. *Cell Cycle* **13**, 2019–2020 (2014).

27. Y. Fujihara, Y. Lu, T. Noda, A. Oji, T. Larasati, K. Kojima-Kita, Z. Yu, R. M. Matzuk, M. M. Matzuk, M. Ikawa, Spermatozoa lacking fertilization influencing membrane protein (FIMP) fail to fuse with oocytes in mice. *Proc. Natl. Acad. Sci. U.S.A.* **117**, 9393–9400 (2020).

28. N. Inoue, T. Kasahara, M. Ikawa, M. Okabe, Identification and disruption of sperm-specific angiotensin converting enzyme-3 (ACE3) in mouse. *PLOS ONE* **5**, e10301 (2010).

29. N. Inoue, I. Wada, Monitoring dimeric status of IZUMO1 during the acrosome reaction in living spermatozoon. *Cell Cycle* **17**, 1279–1285 (2018).

30. L. Hobohm, T. Koudelka, F. H. Bahr, J. Truberg, S. Kapell, S.-S. Schacht, D. Meisinger, M. Mengel, A. Jochimsen, A. Hofmann, L. Heintz, A. Tholey, M. Voss, N-terminome analyses underscore the prevalence of SPPL3-mediated intramembrane proteolysis among Golgi-resident enzymes and its role in Golgi enzyme secretion. *Cell. Mol. Life Sci.* **79**, 185 (2022).

31. S. L. Makowski, Z. Wang, J. L. Pomerantz, A protease-independent function for SPPL3 in NFAT activation. *Mol. Cell. Biol.* **35**, 451–467 (2015).

32. G. P. Otto, D. Sharma, R. S. B. Williams, Non-catalytic roles of presenilin throughout evolution. *J. Alzheimers Dis.* **52**, 1177–1187 (2016).

33. D. Sharma, G. Otto, E. C. Warren, P. Beesley, J. S. King, R. S. B. Williams, Gamma secretase orthologs are required for lysosomal activity and autophagic degradation in *Dictyostelium discoideum*, independent of PSEN (presenilin) proteolytic function. *Autophagy* **15**, 1407–1418 (2019).

34. Z. Zhang, H. Hartmann, V. M. Do, D. Abramowski, C. Sturchler-Pierrat, M. Staufenbiel, B. Sommer, M. van de Wetering, H. Clevers, P. Saftig, B. De Strooper, X. He, B. A. Yankner, Destabilization of β-catenin by mutations in presenilin-1 potentiates neuronal apoptosis. *Nature* **395**, 698–702 (1998).

35. H. Tu, O. Nelson, A. Bezprozvanny, Z. Wang, S.-F. Lee, Y.-H. Hao, L. Serneels, B. de Strooper, G. Yu, I. Bezprozvanny, Presenilins form ER Ca^{2+} leak channels, a function disrupted by familial Alzheimer's disease-linked mutations. *Cell* **126**, 981–993 (2006).

36. I. Dulloo, S. Muliyil, M. Freeman, The molecular, cellular and pathophysiological roles of iRhom pseudoproteases. *Open Biol.* **9**, 190003 (2019).

37. M. Cavadas, I. Oikonomidi, C. J. Gaspar, E. Burbridge, M. Badenes, I. Félix, A. Bolado, T. Hu, A. Bileck, C. Gerner, P. M. Domingos, A. von Kriegsheim, C. Adrain, Phosphorylation of iRhom2 controls stimulated proteolytic shedding by the metalloprotease ADAM17/TACE. *Cell Rep.* **21**, 745–757 (2017).

38. X. Li, T. Maretzky, G. Weskamp, S. Monette, X. Qing, P. D. A. Issuree, H. C. Crawford, D. R. McIlwain, T. W. Mak, J. E. Salmon, C. P. Blobel, iRhoms 1 and 2 are essential upstream regulators of ADAM17-dependent EGFR signaling. *Proc. Natl. Acad. Sci. U.S.A.* **112**, 6080–6085 (2015).

39. C. Adrain, M. Zettl, Y. Christova, N. Taylor, M. Freeman, Tumor necrosis factor signaling requires iRhom2 to promote trafficking and activation of TACE. *Science* **335**, 225–228 (2012).

40. S. Olenic, L. Heo, M. Feig, L. Kroos, Inhibitory proteins block substrate access by occupying the active site cleft of *Bacillus subtilis* intramembrane protease SpoIVFB. *eLife* **11**, e74275 (2022).

41. J.-Y. Hur, G. R. Frost, X. Wu, C. Crump, S. J. Pan, E. Wong, M. Barros, T. Li, P. Nie, Y. Zhai, J. C. Wang, J. TCW, L. Guo, A. McKenzie, C. Ming, X. Zhou, M. Wang, Y. Sagi, A. E. Renton, B. T. Esposito, Y. Kim, K. R. Sadleir, I. Trinh, R. A. Rissman, R. Vassar, B. Zhang, D. S. Johnson, E. Masliah, P. Greengard, A. Goate, Y.-M. Li, The innate immunity protein IFITM3 modulates γ-secretase in Alzheimer's disease. *Nature* **586**, 735–740 (2020).

42. G. He, W. Luo, P. Li, C. Remmers, W. J. Netzer, J. Hendrick, K. Bettayeb, M. Flajolet, F. Gorelick, L. P. Wennogle, P. Greengard, Gamma-secretase activating protein is a therapeutic target for Alzheimer's disease. *Nature* **467**, 95–98 (2010).

43. J. C. Villa, D. Chiu, A. H. Brandes, F. E. Escorcia, C. H. Villa, W. F. Maguire, C.-J. Hu, E. de Stanchina, M. C. Simon, S. S. Sisodia, D. A. Scheinberg, Y.-M. Li, Nontranscriptional role of Hif-1α in activation of γ-secretase and notch signaling in breast cancer. *Cell Rep.* **8**, 1077–1092 (2014).

44. R. D. Teasdale, M. R. Jackson, Signal-mediated sorting of membrane proteins between the endoplasmic reticulum and the Golgi apparatus. *Annu. Rev. Cell Dev. Biol.* **12**, 27–54 (1996).

45. C. Kaether, J. Scheuermann, M. Fassler, S. Zilow, K. Shirotani, C. Valkova, B. Novak, S. Kacmar, H. Steiner, C. Haass, Endoplasmic reticulum retention of the γ-secretase complex component Pen2 by Rer1. *EMBO Rep.* **8**, 743–748 (2007).

46. M. Fassler, M. Zocher, S. Klare, A. G. de La Fuente, J. Scheuermann, A. Capell, C. Haass, C. Valkova, A. Veerappan, D. Schneider, C. Kaether, Masking of transmembrane-based retention signals controls ER export of γ-secretase. *Traffic* **11**, 250–258 (2010).

47. Y. Lu, K. Shimada, J. Zhang, Y. Ogawa, S. Tang, T. Noda, H. Shibuya, M. Ikawa, 1700029I15Rik orchestrates the biosynthesis of acrosomal membrane proteins required for sperm–egg fusion. bioRxiv 488448 [**Preprint**]. 2022; https://doi.org/10.1101/2022.04.15.488448.

48. L. Potocki, L. G. Shaffer, Interstitial deletion of 11(p11.2p12): A newly described contiguous gene deletion syndrome involving the gene for hereditary multiple exostoses (EXT2). *Am. J. Med. Genet.* **62**, 319–325 (1996).

49. H.-G. Kim, H.-T. Kim, N. T. Leach, F. Lan, R. Ullmann, A. Silahtaroglu, I. Kurth, A. Nowka, I. S. Seong, Y. Shen, M. E. Talkowski, D. Ruderfer, J.-H. Lee, C. Glotzbach, K. Ha, S. Kjaergaard, A. V. Levin, B. F. Romeike, T. Kleefstra, O. Bartsch, S. H. Elsea, E. W. Jabs, M. E. MacDonald, D. J. Harris, B. J. Quade, H.-H. Ropers, L. G. Shaffer, K. Kutsche, L. C.

Layman, N. Tommerup, V. M. Kalscheuer, Y. Shi, C. C. Morton, C.-H. Kim, J. F. Gusella, Translocations disrupting PHF21A in the Potocki-Shaffer-syndrome region are associated with intellectual disability and craniofacial anomalies. *Am. J. Hum. Genet.* **91**, 56–72 (2012).

50. A. H. Ligon, L. Potocki, L. G. Shaffer, D. Stickens, G. A. Evans, Gene for multiple exostoses (EXT2) maps to 11(p11.2p12) and is deleted in patients with a contiguous gene syndrome. *Am. J. Med. Genet.* **75**, 538–540 (1998).

51. G. Wennemuth, A. E. Carlson, A. J. Harper, D. F. Babcock, Bicarbonate actions on flagellar and Ca^{2+}−channel responses: Initial events in sperm activation. *Development* **130**, 1317–1326 (2003).

52. N. Mannowetz, S. Kartarius, G. Wennemuth, M. Montenarh, Protein kinase CK2 and new binding partners during spermatogenesis. *Cell. Mol. Life Sci.* **67**, 3905–3913 (2010).

53. K. Hunniger, A. Krämer, M. Soom, I. Chang, M. Köhler, R. Depping, R. H. Kehlenbach, C. Kaether, Notch1 signaling is mediated by importins α3, 4, and 7. *Cell. Mol. Life Sci.* **67**, 3187–3196 (2010).

54. T. Mentrup, K. Theodorou, F. Cabrera-Cabrera, A. O. Helbig, K. Happ, M. Gijbels, A. C. Gradtke, B. Rabe, A. Fukumori, H. Steiner, A. Tholey, R. Fluhrer, M. Donners, B. Schroder, Atherogenic LOX-1 signaling is controlled by SPPL2-mediated intramembrane proteolysis. *J. Exp. Med.* **216**, 807–830 (2019).

55. B. Schröder, C. Wrocklage, A. Hasilik, P. Saftig, Molecular characterisation of "transmembrane protein 192" (TMEM192), a novel protein of the lysosomal membrane. *Biol. Chem.* **391**, 695–704 (2010).

56. C. Zachos, J. Blanz, P. Saftig, M. Schwake, A critical histidine residue within LIMP-2 mediates pH sensitive binding to its ligand β-glucocerebrosidase. *Traffic* **13**, 1113–1123 (2012).

57. T. Mentrup, R. Häsler, R. Fluhrer, P. Saftig, B. Schröder, A cell-based assay reveals nuclear translocation of intracellular domains released by SPPL proteases. *Traffic* **16**, 871–892 (2015).

58. Y. Perez-Riverol, A. Csordas, J. Bai, M. Bernal-Llinares, S. Hewapathirana, D. J. Kundu, A. Inuganti, J. Griss, G. Mayer, M. Eisenacher, E. Pérez, J. Uszkoreit, J. Pfeuffer, T. Sachsenberg, S. Yilmaz, S. Tiwary, J. Cox, E. Audain, M. Walzer, A. F. Jarnuczak, T. Ternent, A. Brazma, J. A. Vizcaíno, The PRIDE database and related tools and resources in 2019: Improving support for quantification data. *Nucleic Acids Res.* **47**, D442-D450 (2019).

59. E. W. Deutsch, N. Bandeira, V. Sharma, Y. Perez-Riverol, J. J. Carver, D. J. Kundu, D. García-Seisdedos, A. F. Jarnuczak, S. Hewapathirana, B. S. Pullman, J. Wertz, Z. Sun, S. Kawano, S. Okuda, Y. Watanabe, H. Hermjakob, B. Maclean, M. J. MacCoss, Y. Zhu, Y. Ishihama, J. A. Vizcaíno, The ProteomeXchange Consortium in 2020: Enabling "big data" approaches in proteomics. *Nucleic Acids Res.* **48**, D1145–D1152 (2020).

60. A. Schlosser, R. Volkmer-Engert, Volatile polydimethylcyclosiloxanes in the ambient laboratory air identified as source of extreme background signals in nanoelectrospray mass spectrometry. *J. Mass Spectrom.* **38**, 523–525 (2003).

61. D. N. Perkins, D. J. C. Pappin, D. M. Creasy, J. S. Cottrell, Probability-based protein identification by searching sequence databases using mass spectrometry data. *Electrophoresis* **20**, 3551–3567 (1999).
